# KDM5 Interacts with Foxo to Modulate Cellular Levels of Oxidative Stress

**DOI:** 10.1371/journal.pgen.1004676

**Published:** 2014-10-16

**Authors:** Xingyin Liu, Christina Greer, Julie Secombe

**Affiliations:** Department of Genetics, Albert Einstein College of Medicine, Bronx, New York, United States of America; Stanford University School of Medicine, United States of America

## Abstract

Increased cellular levels of oxidative stress are implicated in a large number of human diseases. Here we describe the transcription co-factor KDM5 (also known as Lid) as a new critical regulator of cellular redox state. Moreover, this occurs through a novel KDM5 activity whereby it alters the ability of the transcription factor Foxo to bind to DNA. Our microarray analyses of *kdm5* mutants revealed a striking enrichment for genes required to regulate cellular levels of oxidative stress. Consistent with this, loss of *kdm5* results in increased sensitivity to treatment with oxidizers, elevated levels of oxidized proteins, and increased mutation load. KDM5 activates oxidative stress resistance genes by interacting with Foxo to facilitate its recruitment to KDM5-Foxo co-regulated genes. Significantly, this occurs independently of KDM5's well-characterized demethylase activity. Instead, KDM5 interacts with the lysine deacetylase HDAC4 to promote Foxo deacetylation, which affects Foxo DNA binding.

## Author Summary

Reactive oxygen species are essential signaling molecules within the cell. However, when levels of these reactive intermediates become too high (oxidative stress), they cause significant damage to proteins and DNA. It is therefore vitally important to understand how cells regulate genes required to limit oxidative stress. Here we describe a new role for the transcription co-factor KDM5 as an activator of genes that prevent oxidative stress. KDM5 activates these genes by interacting with the transcription factor Foxo and affecting its ability to be recruited to target promoters. Our data provide new insights into the mechanisms by which redox state is regulated, and into the multiple means by which KDM5 regulates gene expression.

## Introduction

Tightly regulated transcription is essential for developmental and homeostatic processes, and is an important means by which animals respond to environmental cues. In addition to sequence-specific transcription factors that activate or repress transcription, an additional layer of gene expression regulation is provided by covalent modifications that occur on the tails of nucleosomal histones such as methylation and acetylation [1]. These chromatin modifications can alter DNA compaction to influence the ability of transcription factors to bind, and form docking sites for additional regulatory proteins that affect gene expression [2]. KDM5 proteins are an important family of transcriptional co-factors because they can both recognize and enzymatically modify specific chromatin modifications [3], [4]. KDM5 proteins are therefore able to regulate gene expression by more than one mechanism leading to context-dependent activation or repression of transcription.

Utilizing their well-characterized Jumonji (JmjC) domain, KDM5 proteins function as lysine demethylases [3]–[5]. The four mammalian KDM5 paralogs can demethylate di- and trimethylated histone H3 lysine 4 (H3K4me2/3) while the sole *Drosophila* KDM5 ortholog specifically targets H3K4me3 [6]–[13]. Because H3K4me2/3-containing nucleosomes are primarily found at promoter regions and are a hallmark of actively transcribed genes, removal of these chromatin marks by KDM5 demethylases correlates with transcriptional repression [14]. Interestingly, while *Drosophila* KDM5 and mouse KDM5B are essential genes, the demethylase function of these proteins is dispensable for viability [15]–[17]. This finding highlights the importance of the other gene-regulatory activities of KDM5 proteins. Indeed, KDM5 family proteins can influence gene expression through changes to histone acetylation by interacting with lysine deacetylases such as HDAC1 and HDAC4 [18]–[20]. In addition, KDM5 proteins can bind to unmodified histone H3 and histone H3 that is methylated on lysine 4 via their PHD finger motifs, although the biological importance of these chromatin-recognition activities remains largely uncharacterized [15], [21].

Emphasizing the importance of their gene-regulatory functions, fly KDM5 and mouse KDM5B are essential for viability [17], [22]. Moreover, three of the four human paralogs are implicated in disease. KDM5A and KDM5B are overexpressed in a large number of tumors including melanoma, breast, gastric and lung cancer (reviewed by [3]). While the precise role of KDM5A and KDM5B in tumor formation remains to be elucidated, recent evidence supports the interesting notion that KDM5B overexpression promotes the growth and survival of cancer “stem cells” [23], [24]. These oncogenic functions may be explained in part by the observation made by us and others that KDM5 family proteins interact with the well-known oncoprotein Myc and the tumor suppressor pRB [13], [15], [25]–[28]. Unlike dysregulation of KDM5A and KDM5B that are linked to cancer, mutations in KDM5C are found in patients with syndromic and non-syndromic intellectual disability [29]–[32]. This suggests that this family member plays an essential role in neuronal development. Consistent with this, KDM5C is expressed at high levels in neuronal cells and its knockdown in cultured rat cerebellar neurons causes dendritic defects [8]. KDM5 family proteins are therefore of central importance for normal cellular growth and function in a range of cell types.

Here we describe KDM5 as a new regulator of cellular levels of oxidative stress. Moreover, this occurs by a previously undescribed mechanism in which KDM5 influences transcription factor binding. Our data demonstrate that *Drosophila* KDM5 directly activates genes required to regulate cellular redox state. Consistent with this, *kdm5* mutants are sensitive to treatment with the oxidizer paraquat, show elevated levels of oxidized proteins, and have an increased mutation load. Significantly, KDM5 interacts with the well-established oxidative stress transcription factor Foxo and is required for its recruitment to target promoters. These data expand the repertoire of KDM5's gene-regulatory functions and significantly add to our understanding of how transcription factors find and bind to their cognate DNA binding sites *in vivo*. Because increased cellular oxidative stress is implicated in cancer and neuronal dysfunction [33]–[35], our data have clear implications for understanding how dysregulation of KDM5 family proteins results in human disease.

## Results

### *kdm5* mutants have deregulated expression of genes required for oxidative stress resistance

**Figure 1:**
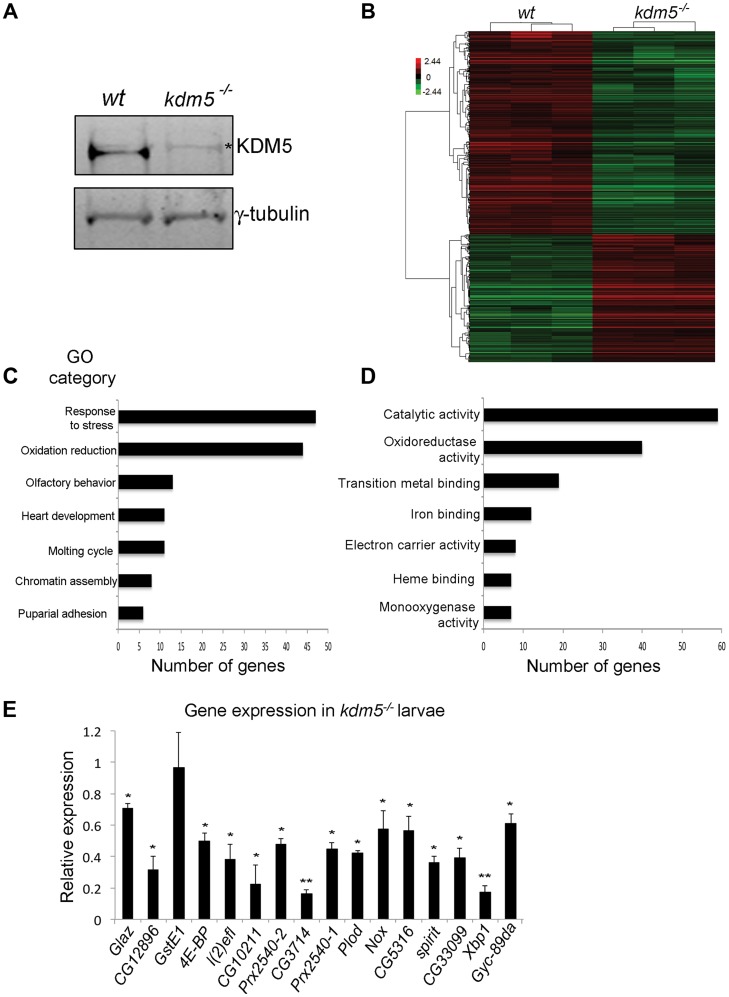
KDM5 mutants show reduced expression of genes required to reduce oxidative stress. A) Western blot showing levels of KDM5 and the loading control γ-tubulin in wildtype (*w^1118^*) and *kdm5^10424^* homozygous mutant wing imaginal discs used for microarrays. * indicates a non-specific band. (B) Hierarchical clustering analysis of 901 genes that are differentially expressed 1.5-fold or more in *kdm5* mutant wing discs compared to wildtype (*w^1118^*) (*p*<0.05). Up-regulated genes are shown in red; down-regulated genes in green. (C) Gene ontology analyses showing biological process enrichment analysis of down- and up-regulated genes (*p*≤0.01). (D) GO David analyses of the Molecular function of the 84 stress response and oxidation reduction class genes (*p*≤0.01). (E) Real-time PCR showing levels of oxidative stress response genes in *kdm5^10424/K6801^* 3^rd^ instar female larvae relative to wildtype. Levels of gene expression within each sample were normalized to *rp49* and shown relative to wildtype. * *p*<0.05, ** *p*<0.01.

To determine the gene expression changes associated with loss of KDM5, we carried out microarray analyses of dissected wing imaginal discs from wildtype (*w^1118^*) and *kdm5^10424^* homozygous mutant 3^rd^ instar larvae. *kdm5^10424^* behaves genetically as a strong loss of function allele and we are unable to detect KDM5 protein by Western blot (Figure 1A). These analyses revealed that of the 18,500 transcripts represented on the array, 367 genes were up-regulated and 534 genes were down-regulated 1.5-fold or more in *kdm5* mutants compared to wildtype (*p*<0.01; Figure 1B; Figure S1A–F). A majority of genes up or down-regulated in *kdm5* mutant wing discs were moderately affected (1.5 to 5-fold), suggesting that KDM5 does not cause dramatic changes to gene expression levels (Figure S1G). Using real time-PCR we confirmed the expression of five downregulated and five upregulated transcripts, validating our microarray data (Figure S1H).

To highlight biological processes regulated by KDM5, we determined which gene ontology (GO) terms were enriched within the differentially expressed genes (both up and downregulated). While a total of ten GO terms were identified, two functionally related and overlapping terms had the largest number of genes: (1) response to stress and (2) oxidation reduction (Figure 1C). These two classes had 45 and 44 genes, respectively, with 7 that overlap. 45 of these 82 stress response and oxidation reduction genes were downregulated and 37 were upregulated (Figure 1D; Figure S2). Downregulated genes include those with established roles in responding to oxidative and other forms of stress. For example the translation initiation factor 4E-BP that, while also involved in other processes, also regulates the translation of components of mitochondrial complex I and is required for survival in conditions of oxidative stress [36]–[38]. In addition, *kdm5* mutants show downregulation of peroxiredoxins that detoxify H_2_O_2_ (e.g. *Prx2540-1*, *Prx2540-2*, *CG12896*, *CG10211*) [39]–[41], maintain cellular NAD levels (*CG3714*) [42], or function as chaperones (*Glaz*, *l(2)efl*) [43], [44]. In contrast, upregulated genes include *methuselah* (*mth*) family genes that negatively regulate oxidative stress resistance [45]. These data suggest that KDM5 may be a new regulator of cellular redox state.

To understand the role of KDM5 in the transcriptional regulation of oxidative stress resistance, we focused on 16 genes involved in this process that were downregulated in our *kdm5* mutant microarray data. These genes require KDM5 for their activation, and the mechanisms by which KDM5 induces gene expression are not well characterized. To examine their expression in whole 3^rd^ instar larvae we used animals homozygous for *kdm5^10424^* or transheterozygous for the allelic combination of *kdm5^10424^* and *kdm5^K06801^* (*kdm5^10424/K06801^*). All *kdm5* mutants examined showed reduced expression of 15 out of the 16 genes tested (Figure 1E). While one gene (*GstE1*) was significantly downregulated in wing imaginal discs, it was not reduced in whole larvae, suggesting that its regulation by KDM5 is tissue specific. KDM5 is therefore required to maintain the expression of oxidative stress resistance genes in many other larval tissues in addition to wing disc cells where this regulation was first identified.

### KDM5 is required for survival in conditions of oxidative stress

**Figure 2:**
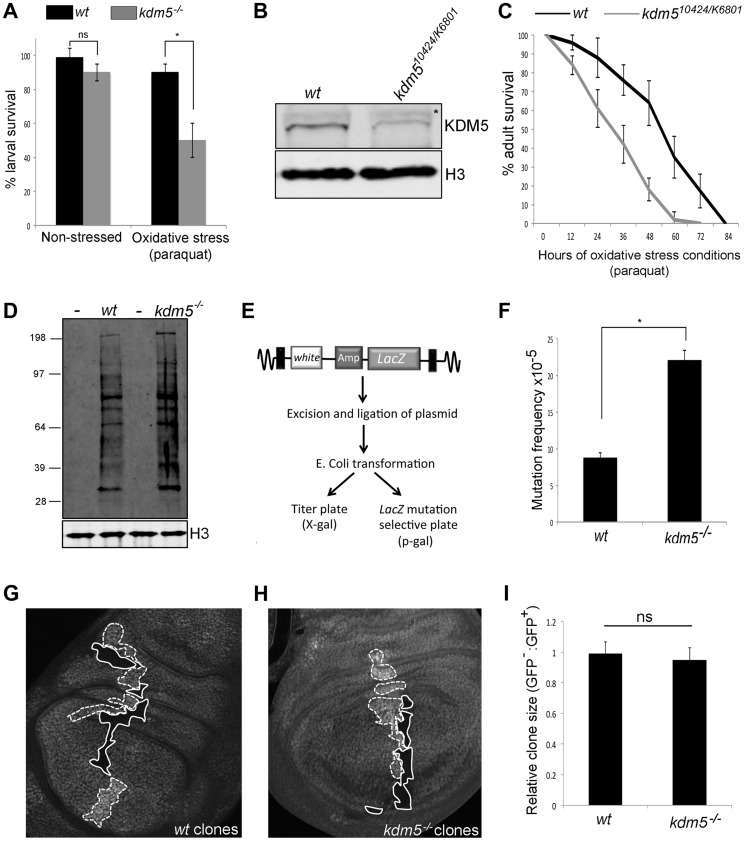
Reducing KDM5 affects cellular levels of oxidative stress. (A) Survival of wildtype control (*w^1118^*) and *kdm5^10424/K06801^* female larvae in 5% sucrose (left) or in 20 mM paraquat/5% sucrose (right) for six hours. * *p*<0.01. (B) Western blot showing levels of KDM5 and the loading control histone H3 in wildtype (*w^1118^*) and *kdm5^10424/K6801^* adult female heads (three heads per lane). (C) Survival curve of *w^1118^* and *kdm5^10424/K06801^* adult females fed 20 mM paraquat in 5% sucrose. Adults were one to three days old at the start of the experiment. Error bars represent standard error. The two survival curves are significantly different from one another (*p*<0.05). (D) Detection of oxidized proteins in wildtype and *kdm5^K6801^* homozygous mutant 3^rd^ instar larvae using oxyblot. Molecular weight markers are shown on the left. – indicates negative control lanes in which carbonyl groups were not derivatized. *kdm5* mutants show ∼2-fold increase in total levels of oxidized proteins. Histone H3 is shown as a loading control. (E) Schematic of *lacZ* mutation reporter assay. Adapted from Garcia et al [51]. (F) *lacZ* mutation frequency of wildtype control (*w^1118^*) and *kdm5^10424^* homozygous mutant 3^rd^ instar larvae. Genotypes are *w^1118^*; #9lacZ; + (labeled *wt*) and *w^1118^*; *kdm5^10424^*, #9lacZ; + (labeled *kdm5^−/−^*. * *p*<0.01. (G) Generation of wildtype clones in a wildtype background in larval wing imaginal disc. Genotype is hs-FLP^122^/+; FRT40A/FRT40A ubi-GFP. Areas circled with dashed line have two copies of ubi-GFP (twin spots). Areas shown with a solid line have no GFP. (H) Generation of *kdm5^K6801^* mutant clones in wing imaginal disc. Genotype is hs-FLP^122^/+; *kdm5^K6801^* FRT40A/FRT40A ubi-GFP. Areas shown with a dashed line have two copies of GFP (twin spots). Areas circled with sold line are homozygous mutant for *kdm5^K6801^*. (I) Quantitation of clone area in E (*wt*; N = 6) and F (*kdm5* mutant; N = 8). ns = not significantly different.

Because mutations in several genes that require KDM5 for their activation show sensitivity to conditions of oxidative stress (e.g. *4E-BP*, *Glaz*, *l(2)efl*; [37], ), we tested whether *kdm5* mutant larvae were sensitive to treatment with the established oxidizer paraquat. While *kdm5* mutant larvae survive in a similar manner to wildtype in non-stressed conditions, they die at a significantly higher rate when exposed to paraquat for six hours (Figure 2A). KDM5 is therefore required for larval survival to conditions of acute oxidative stress.

We also tested whether adult flies with reduced levels of KDM5 were sensitive to conditions of oxidative stress. To do this, we used adult flies that were transheterozygous for *kdm5^10421^* and *kdm5^K06801^*. While *kdm5^10424^* and *kdm5^K06801^* are both homozygous lethal [22], combining these alleles decreases KDM5 protein levels 70–80% and adults eclose at 50% of the expected frequency (Figure 2B; Table S1) [13], [15]. As shown in Figure 2C and Figure S3A for females and males, respectively, *kdm5^10424/K06801^* mutant adults die significantly faster than wildtype controls when treated with paraquat. *kdm5* mutant larvae and adults therefore show similar oxidative stress-sensitivity phenotypes. It should be noted that although *kdm5^10424/K06801^* adults survive to the same degree as wildtype for the first five days in non-stressed conditions, these animals are shorter-lived than wildtype (Figure S3B, C). To confirm a role for KDM5 in oxidative stress resistance, we used adult-specific RNAi-mediated knockdown, as this decreases lifespan in non-stressed conditions by only 7% compared to 87% for *kdm5^10424/K6801^* (Figure S4A, B). Because ubiquitous expression of KDM5 RNAi transgenes throughout development causes lethality, we combined Actin-Gal4 with a ubiquitously expressed temperature sensitive Gal4 inhibitor Gal80^TS^ to control transgene expression (Gal80^TS^; Act^TS^ when combined) [47]. By crossing KDM5 RNAi transgenes (or a control GFP RNAi transgene) at 18°C, transgene expression is kept off, but is activated by shifting adults to 25°C. Like *kdm5^10242/K6801^* mutants, ubiquitous expression of a KDM5 RNAi transgene during adulthood resulted in sensitivity to paraquat (Figure S4C). Control adults maintained at 18°C, at which temperature the RNAi transgene is not expressed, did not show this phenotype (Figure S4D, E). Because mutations in other genes (e.g. Foxo and Nrf2) [48], [49] that cause sensitivity to oxidative stress also affect sensitivity to other stressors, we also tested whether KDM5 knockdown adults were sensitive to treatment with a xenobiotic. Adult specific RNAi-mediated knockdown of KDM5 resulted in sensitivity to the well-characterized insecticide DDT (dichloro-diphenyl-trichloroethane) (Figure S4F). KDM5 is therefore essential for survival in response to environmental conditions of oxidative and xenobiotic stress.

### Reducing KDM5 elevates levels of oxidized proteins and increases mutation frequency

Increased cellular levels of oxidative stress can cause cellular dysfunction by oxidizing proteins and DNA [50]. We therefore examined overall levels of protein oxidation in *kdm5* mutant larvae and found that they have ∼2-fold increase in the levels of carbonyl groups that are introduced into proteins by oxidative reactions (Figure 2D). We also tested whether *kdm5* mutant larvae have an increased nuclear DNA mutation frequency using a *lacZ* mutation transgene (Figure 2E) [47], [51]. Using this reporter, we found that *kdm5* mutant larvae have a 2-fold higher mutation frequency than genetic background-matched controls (Figure 2F). Cells lacking KDM5 therefore experience increased oxidative stress even in the absence of any exogenous source of stress and this is toxic to both proteins and DNA. Importantly, *kdm5* mutant larvae occur at approximately at the expected Mendelian ratio and *kdm5* mutant cells do not show any overt growth phenotype (Figure 2G–I). The phenotypes we observe in *kdm5* mutant larvae are therefore unlikely to be downstream consequences to growth and/or proliferation defects.

### KDM5 physically and genetically interacts with Foxo, an established oxidative stress response transcription factor

**Figure 3:**
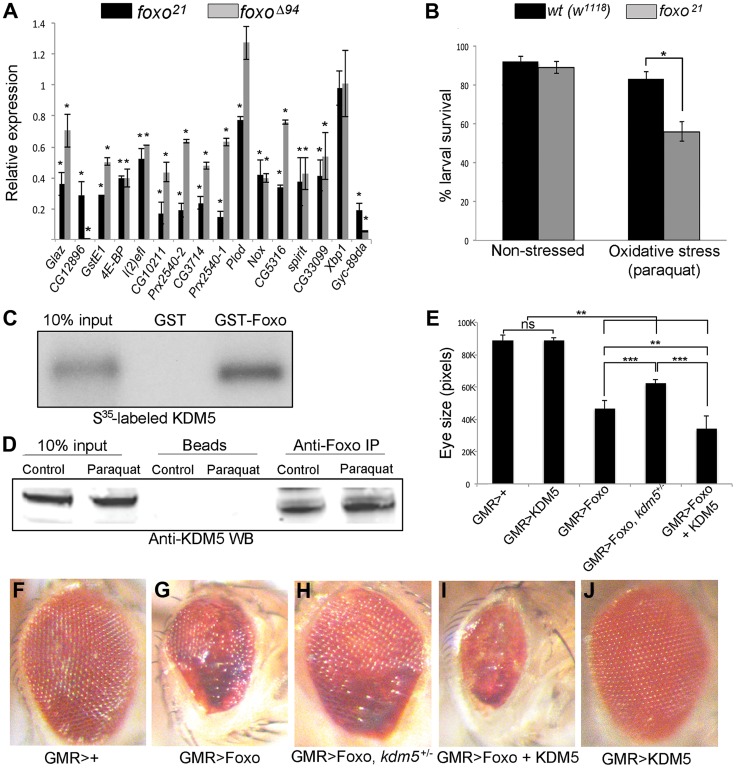
KDM5 and Foxo regulate a common set of genes and genetically and physically interact. (A) Real-time PCR showing levels of gene expression in *foxo^21^* or foxo^Δ94^ homozygous 3^rd^ instar female larvae relative to wildtype controls (*w^1118^*). *foxo^21^* was maintained as a homozygous stock while *foxo^Δ94^* was generated by intercrossing heterozygous parents, so potentially contains some maternally derived Foxo. * *p*<0.05. (B) Survival of wildtype (*w^1118^*) and *foxo^21^* female larvae in 5% sucrose (left) or with 20 mM paraquat in 5% sucrose (right) for six hours. (C) *In vitro* binding assay showing that S^35^-labeled full length KDM5 binds to GST-Foxo but not to GST alone. (D) *in vivo* interaction between Foxo and KDM5. Foxo protein was immunoprecipitated from S2 cell nuclear extract from cells grown in vehicle or oxidative stress conditions (20 mM paraquat for six hours). Levels of KDM5 in Foxo immunoprecipitates were then examined by Western blot and compared to beads alone. (E) Quantitation of genetic interaction between *kdm5* and *foxo* using total eye area (>10 eyes per genotype) as measured in pixels using ImageJ. Error bars indicate standard deviation. Significance was determined using a Student's t-test. ** *p*<0.001, *** *p*<0.0001. ns = not significant. (F–J) Representative images of the genetic interaction between Foxo and KDM5 in 15 day old flies. (F) Control flies are GMR-Gal4 crossed to wildtype (*w^1118^*) and (G) GMR-Gal4 combined with UAS-Foxo showing rough eye phenotype. (H) GMR-Gal4 combined with UAS-Foxo and heterozygous for *kdm5^K6801^* showing suppression of GMR>Foxo eye phenotype. (I) GMR-Gal4 combined with UAS-Foxo and UAS-KDM5 showing eye phenotype enhancement. (J) GMR-Gal4 crossed to UAS-KDM5.

The 16 oxidative stress genes shown in Figure 1E all have binding site(s) for Foxo, which is a transcription factor known to be integral to responding to conditions of oxidative stress [52]. One gene, *4E-BP* is a well-established direct Foxo target gene [53], [54]. The remaining genes are candidate Foxo targets based on their downregulation in *foxo* mutant larvae microarrays or being bound by Foxo binding using ChIP-chip from wildtype larvae or ChIP-seq from adults [37], [55]–[57]. To confirm that these were Foxo regulated genes, we examined their expression in *foxo^21^* and *foxo^Δ94^* homozygous mutant larvae and found that 14 of the 16 genes tested were downregulated in both mutant strains (Figure 3A). Thus 13 oxidative stress sensitivity genes tested showed similar downregulation in *kdm5* and *foxo* mutant larvae. In a similar manner to *kdm5* mutant and knockdown adults, *foxo* mutant flies have been shown to be sensitive to conditions of oxidative stress [53] (Figure S5A). Further confirming their similarities, *kdm5* and *foxo* mutant larvae display similar rates of death when subjected to paraquat-mediated oxidative stress (Figure 3B).

Based on the gene expression and phenotypic similarities caused by loss of KDM5 and Foxo, we tested whether these two proteins form a complex. *In vitro* transcribed and translated S^35^-labeled KDM5 bound robustly to GST-Foxo but did not bind to GST alone, demonstrating that these proteins physically interact (Figure 3C; Figure S5B). KDM5 and Foxo also interact *in vivo*, as immunoprecipitating Foxo from S2 cell nuclear extracts co-precipitated KDM5 in non-stressed cells and in cells subjected to increased oxidative stress through treatment with paraquat (Figure 3D; Figure S5C).

To assess the general requirement for KDM5 in Foxo function, we tested whether they genetically interact using an adult eye phenotype induced by overexpression of Foxo (Figure 3E–J). GMR-Gal4-mediated overexpression of Foxo causes an adult eye phenotype due to a reduced cell number and cell size [53], [58] (Figure 3F, G). This eye phenotype was suppressed by genetically reducing KDM5 levels using *kdm5* heterozygotes (*kdm5^K06801^* or *kdm5^10424^*) (Figure 3H). Conversely, co-overexpressing Foxo and KDM5 resulted in a more severe eye phenotype than Foxo alone (Figure 3I). KDM5 overexpression alone did not result in any detectable adult phenotype, nor did *kdm5* heterozygous flies (Figure 3J) [13]. These data for the first time demonstrate that KDM5 is required for Foxo function *in vivo*.

### KDM5 primarily affects the level of oxidative stress genes in non-stressed conditions

The sensitivity of *kdm5* mutants to conditions of oxidative stress may be due to their already reduced expression of oxidative stress resistance genes. KDM5 may also be required for the activation of these genes in response to acute oxidative stress conditions. For these analyses, we focused on 13 genes that were downregulated in *kdm5* and *foxo* mutant larvae. We first tested wildtype larvae to determine which of these genes was activated in response to paraquat treatment. Eight of the 13 genes tested were induced by paraquat in wildtype larvae (Figure S5D), and we further characterized the role of the KDM5/Foxo complex in the regulation of six of these that showed the most consistent activation.

**Figure 4:**
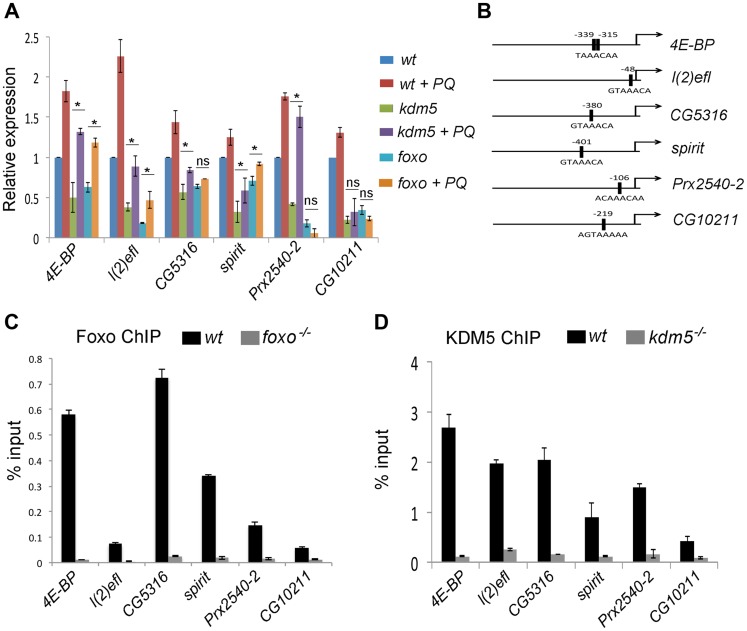
KDM5 directly regulates oxidative stress resistance genes. (A) Real-time PCR analyses of *4E-BP*, *l(2)efl*, *CG5316*, *spirit*, *Prx2540-2* and *CG10211* from wildtype (*w^1118^*), *kdm5^K6801^* mutant and *foxo^21^* mutant 3^rd^ instar larvae in 5% sucrose or 20 mM paraquat/5% sucrose for six hours (oxidative stress). (B) Schematic of the promoters of *4E-BP*, *l(2)efl*, *CG5316*, *spirit*, *Prx2540-2* and *CG10211* showing the position of the Foxo binding site (FHREs; black boxes). Primers surrounding these sites were used for ChIP analyses shown in parts C and D. (C) ChIP analyses of *4E-BP*, *l(2)efl*, *CG5316*, *spirit*, *Prx2540-2* and *CG10211*. Anti-Foxo ChIP is shown in black bars while control anti-Foxo ChIP from *foxo^21^* homozygous mutant larvae is shown in grey. Data are shown as % of input DNA. All six genes tested show significant attenuation of anti-Foxo ChIP signal (*p*<<0.01). (D) ChIP analyses of *4E-BP*, *l(2)efl*, *CG5316*, *spirit*, *Prx2540-2* and *CG10211*. Anti-KDM5 ChIP is shown in black bars while control anti-KDM5 ChIP from *kdm5^K6801^* homozygous mutant larvae is shown in grey. Data are shown as % of input DNA. All six genes tested show significant attenuation of anti-KDM5 ChIP signal (*p*<<0.01).

To determine the requirement for KDM5 and Foxo for stress-mediated gene activation we treated wildtype, *kdm5* and *foxo* mutant larvae with paraquat and examined the expression of KDM5-Foxo target genes (Figure 4A). These data revealed that the primary role for KDM5 and Foxo is in maintaining the endogenous expression of oxidative stress resistance genes and not their exogenous oxidative-stress mediated activation. Three genes, *4E-BP*, *l(2)efl* and *spirit*, were activated in response to paraquat but to lower maximal levels of expression in *kdm5* and *foxo* mutants. KDM5 and Foxo are therefore required for the baseline expression of these genes, but are not essential for their activation in response to stress conditions. Conversely, one gene (*CG10211*) was not activated in paraquat-treated *kdm5* or *foxo* mutants, thus this gene requires KDM5 and Foxo for its expression in both non-stressed and stressed conditions. Interestingly, two genes, the peroxiredoxin *Prx2540-2* and the predicted DNA repair enzyme CG5316 were activated by paraquat in *kdm5* mutant larvae but not in *foxo* mutants. This suggests that while KDM5 and Foxo are required for endogenous levels of expression, only Foxo is required for their paraquat-mediated activation.

### KDM5 and Foxo bind to the same promoter region of co-regulated genes

Based on the gene expression similarities and the genetic and physical interaction between KDM5 and Foxo, we predicted that these proteins act together to regulate gene expression. To test this, we used ChIP to confirm that Foxo bound to the predicted Forkhead response element (FHRE) region within the promoters of six oxidative stress resistance genes in larvae (Figure 4B). Foxo bound to the FHRE promoter region of all six genes using two independent anti-Foxo antibodies and using *foxo* mutant larvae and IgG alone as a negative controls (Figure 4C; Figure S6A, B). It should be noted that while the Foxo ChIP signal to the FHRE regions of *l(2)efl* and *CG10211* are relatively weak (0.1% input), they are significantly attenuated in *foxo* mutant larvae, demonstrating that this signal is Foxo-specific. Consistent with KDM5 functioning with Foxo at these promoters, anti-KDM5 ChIP showed that KDM5 binds to the same FHRE regions as Foxo in wildtype larvae but not *kdm5* mutants (Figure 4D). Importantly, Foxo and KDM5 ChIP analyses of control upstream and downstream regions of these genes revealed background levels of binding that were unaltered in *foxo* or *kdm5* mutants, respectively (Figure S7A–C). To independently confirm KDM5 binding, we used a genomic rescue strategy to generate an epitope-tagged form of KDM5. This HA-tagged form of KDM5 is expressed at endogenous levels and rescues *kdm5* mutants in a similar manner to a wildtype non-tagged version of this transgene [15] (Figure S8A, B). Like our analyses using anti-KDM5, anti-HA ChIP showed enrichment to the FHRE region of target promoters compared to an IgG specificity control (Figure S8C–G).

### KDM5 is required for efficient Foxo promoter recruitment

**Figure 5:**
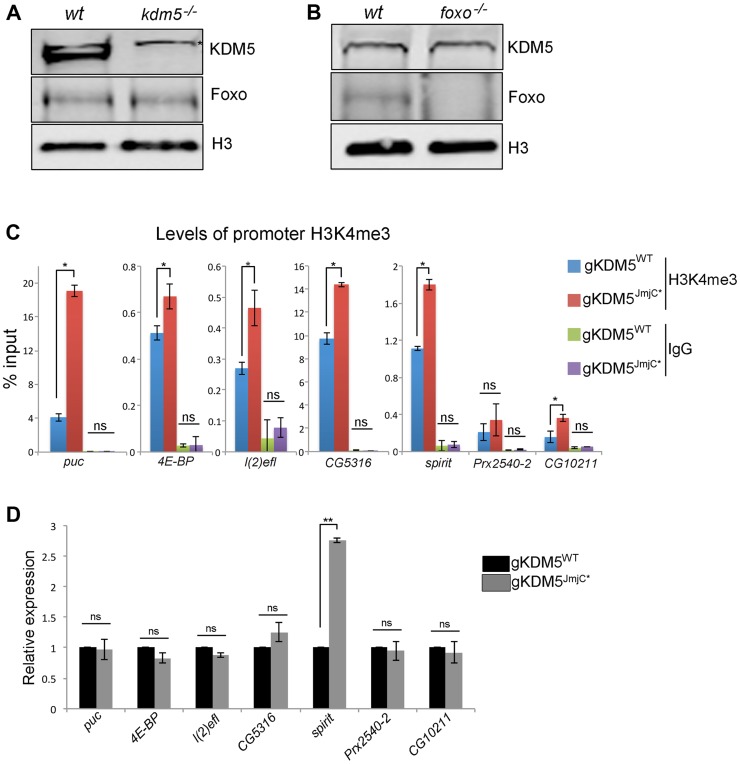
The H3K4me3 demethylase activity of KDM5 is not required for it to regulate KDM5-Foxo target genes. (A) Western blot showing levels of KDM5, Foxo and the loading control histone H3 in wildtype (*w^1118^*) and *kdm5* mutant (*kdm5^K6801^*) 3^rd^ instar wing imaginal discs (10 discs per lane). (B) Western blot showing levels of KDM5, Foxo and histone H3 in wildtype (*w^1118^*), and *foxo^21^* mutant 3^rd^ instar wing discs. (C) H3K4me3 ChIP to the FHRE region of *puc*, *4E-BP*, *l(2)efl*, *CG5316*, *spirit*, *Prx2540-2* and *CG10211* promoters in *lid^10424^*/*lid^10424^*; gKDM5/gKDM5 (blue) and *lid^10424^*/*lid^10424^*; gKDM5^JmjC*^/gKDM5^JmjC*^ (red) larvae. * *p*<0.05. IgG is included as a negative control. (D) Real-time PCR showing expression levels in wildtype (*lid^10424^*/*lid^10424^*; gKDM5/gKDM5 (black) and *lid^10424^*/*lid^10424^*; gKDM5^JmjC*^/gKDM5^JmjC*^ (grey) 3^rd^ instar larvae. Data are normalized to *rp49* expression and changes to expression in demethylase inactive larvae are shown relative to *lid^10424^*/*lid^10424^*; gKDM5/gKDM5 control larvae. ** *p*<0.01.

To address the mechanism by which loss of KDM5 affects Foxo-regulated oxidative stress resistance genes, we first asked whether it was due to alterations in abundance. As shown in Figure 5A and B, levels of Foxo and KDM5 are not altered in *kdm5* and *foxo* mutants, respectively. The changes to gene expression observed are therefore not simply due to the absence of these proteins. We next tested whether KDM5's well-established H3K4me3-directed demethylase activity played a role in the regulation of oxidative stress resistance genes. To do this, we used our previously generated fly strain that specifically lacks KDM5-dependent demethylase activity [15]. Consistent with our previous observation that global levels of H3K4me3 are increased in demethylase-inactive KDM5 mutants, the non-KDM5 target *puc* and the KDM5 target genes *4E-BP*, *l(2)efl*, *CG5316*, *spirit* and *CG10211* all showed increased promoter proximal H3K4me3 (Figure 5C). *Prx2540-2* was the only gene examined that did not show increased levels of H3K4me3, suggesting that not all promoters are equally affected by the loss of KDM5-dependent demethylase activity. Despite increased levels of H3K4me3 that is associated with actively transcribed genes, six of seven genes were expressed at wildtype levels in KDM5 demethylase inactive larvae (Figure 5D). The one gene with increased expression (*spirit*) may be particularly sensitive to increased levels of H3K4me3 (Figure 5D). These data show that the demethylase activity of KDM5 is not required for the activation of KDM5-Foxo oxidative stress target genes. These expression data are consistent with our previous observation that, unlike KDM5 knockdown flies, demethylase inactive flies are not sensitive to conditions of oxidative stress [15].

One means by which KDM5 is known to activate transcription is by inhibiting HDAC1 to increase levels of promoter histone H3 acetylation [18], [19]. To determine if KDM5-mediated HDAC1 inhibition plays a role in the activation of KDM5-Foxo target genes, we tested whether promoter proximal histone H3 acetylation was altered. As shown in Figure S6C, *kdm5* mutant larvae have unaltered levels of histone H3 acetylation at four targets and slightly elevated at the other two. Because KDM5-Foxo targets did not show a consistent pattern of histone acetylation, these genes are likely to be regulated in an HDAC1-independent manner.

**Figure 6:**
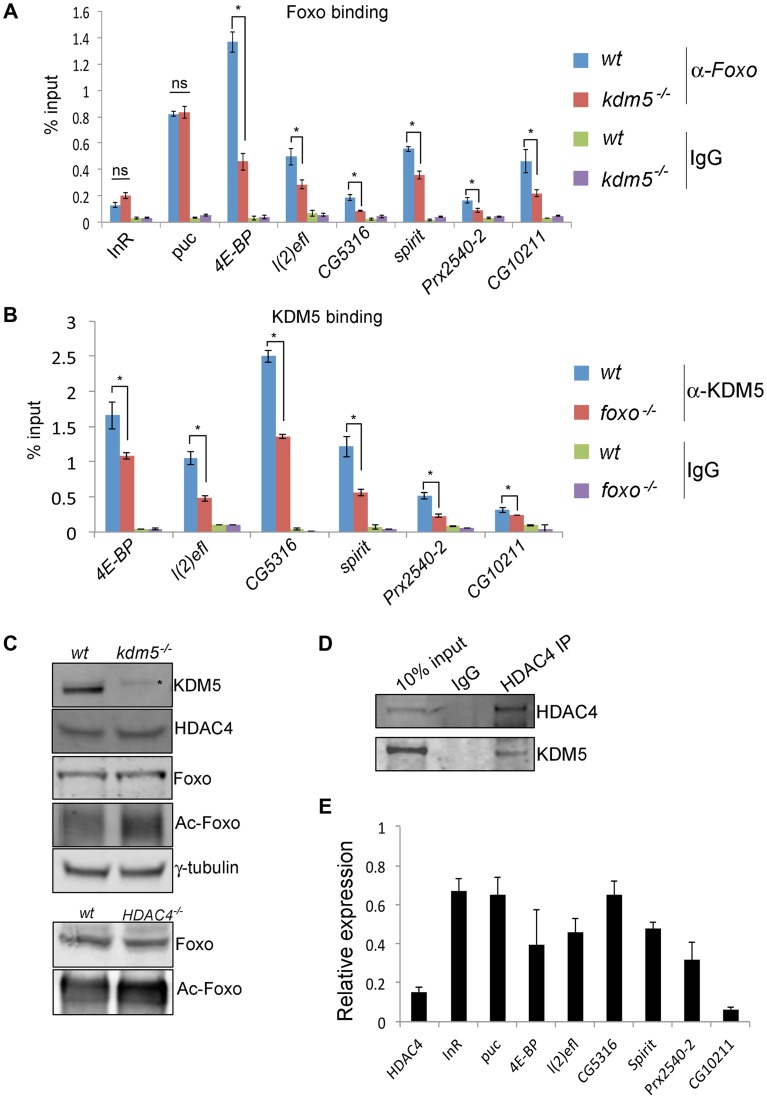
KDM5 is required for efficient recruitment of Foxo to a subset of its target promoters. (A) ChIP using anti-Foxo [86] to the FHRE region of the *InR*, *puc*, *4E-BP*, *l(2)efl*, *CG5316*, *spirit*, *Prx2540-2* and *CG10211* promoters in wildtype (*w^1118^*) and *kdm5^10424/K6801^* mutant larvae. * *p*<0.05. (B) KDM5 ChIP to the FHRE in the *InR*, *puc*, *4E-BP*, *l(2)efl*, *CG5316*, *spirit*, *Prx2540-2* and *CG10211* promoters in wildtype (*w^1118^*) and *foxo^21^* mutant 3^rd^ instar larvae. * *p*<0.05. IgG is included as an additional control for non-specific binding in A and B. (C) (top) Levels of KDM5, HDAC4, Foxo, acetylated Foxo and the loading control γ-tubulin in wildtype and *kdm5^K6801^* mutant larvae (anterior half of 3^rd^ instar larvae). Levels of acetylated Foxo were determined by immunoprecipitating Foxo from larval extract and probing Western blot with an anti-acetyl-lysine antibody. * indicates non-specific band in KDM5 blot. (bottom) Levels of Foxo and acetylated Foxo in *HDAC4^KG09091^* mutant larvae. (D) Immunoprecipitating HDAC4 from larval extracts efficiently pulls down HDAC4 and also co-precipitates KDM5. Lysate used was from 3^rd^ instar larvae. (E) Real time PCR showing levels of *HDAC4*, *InR*, *puc*, *4E-BP*, *l(2)efl*, *CG5316*, *spirit*, *Prx2540-2* and *CG10211* mRNA in *HDAC4^KG09091^* homozygous 3^rd^ instar larvae showing that all of these genes are significantly downregulated. All genes shown were significantly downregulated (*p*<0.05).

We next tested whether KDM5 is required for Foxo promoter recruitment. Significantly, Foxo binding to the six KDM5-Foxo co-regulated genes was attenuated in *kdm5* mutant larvae using two independent anti-Foxo antibodies (Figure 6A and Figure S6B). This is not because Foxo was generally unable to bind DNA in *kdm5* mutants, as its binding to two non-KDM5-regulated target promoters, *InR* and *puc*, was not affected. We also found that KDM5 promoter binding was reduced in *foxo* mutant larvae (Figure 6B). KDM5 and Foxo are therefore reciprocally required for recruitment to the promoters of KDM5-Foxo co-regulated genes.

**Figure 7:**
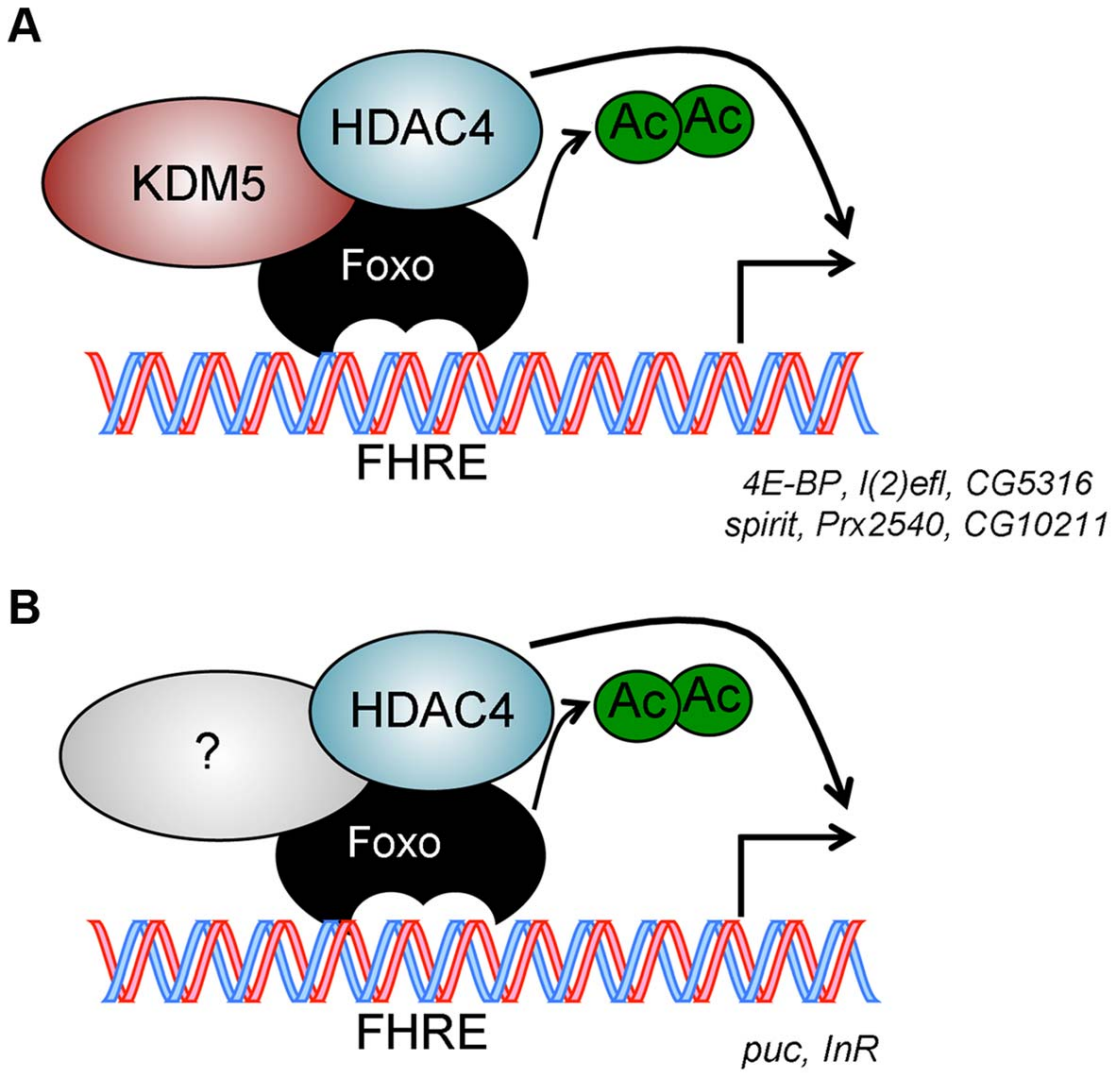
Model for KDM5 function. (A) KDM5-Foxo co-regulated genes are a subset of all Foxo regulated genes. We propose that at these stress response-related promoters, KDM5 facilitates the deacetylation of Foxo (and activation of its DNA binding activity) by binding to HDAC4. (B) Because HDAC4 is required at additional Foxo-regulated genes that are not affected by loss of KDM5, we propose that HDAC4-Foxo complexes that lack KDM5 regulate different targets than KDM5-HDAC4-Foxo complexes (e.g. *InR* and *puc*). Additional, as yet uncharacterized, proteins (shown as unlabeled shapes in A and B) are likely to be present in both KDM5-HDAC4-Foxo and HDAC4-Foxo complexes. See text for details.

Because Foxo DNA binding can be inhibited by acetylation [59], we tested whether KDM5 was a novel effector of this posttranslational modification. While wildtype and *kdm5* mutant larvae had similar overall levels of Foxo, levels of acetylated Foxo were increased (Figure 6C, Figure S9). In both flies and mammalian cells HDAC4 can deacetylate Foxo [60], [61]. Because *kdm5* mutant larvae have wildtype levels of HDAC4, the elevated levels of Foxo acetylation in these animals was not simply due to a deficiency in this enzyme (Figure 6C; Figure S9). We therefore tested whether HDAC4 interacts with KDM5, as human HDAC4 and KDM5B have been previously observed to form a complex [20]. Immunoprecipitating HDAC4 from larval extract co-precipitated KDM5, demonstrating that these proteins interact *in vivo* (Figure 6D; Figure S9). As previously observed [44], larvae homozygous for the *HDAC4^KG09091^* hypomorphic mutation show increased levels of acetylated Foxo (Figure 6C). Moreover, we find that these HDAC4 mutant larvae show decreased expression of KDM5-Foxo target genes, consistent with HDAC4 functioning at these promoters with KDM5/Foxo (Figure 6E). Significantly however, we find that reducing levels of HDAC4 also decreases the expression of the Foxo targets *InR* and *puc* that are not KDM5-regulated (Figure 6E). These data suggest that KDM5 may be a component of a subset of all HDAC4-Foxo complexes that affects acetylation and promoter binding to a subset of target genes (Figure 7).

## Discussion

Here we demonstrate a new function for KDM5 whereby it influences cellular levels of oxidative stress, at least in part by affecting Foxo transcription factor recruitment to a subset of its target promoters. Based on microarray data carried out on *kdm5* mutant wing imaginal discs, we found that KDM5 transcriptionally regulates the expression of a number of genes implicated in regulating cellular redox state. The dysregulation of oxidative stress resistance genes in the absence of KDM5 correlates with elevated levels of oxidized proteins and increased mutation frequency. In addition, reducing levels of KDM5 in larvae or adults resulted in increased sensitivity to environmental oxidizers such as paraquat. Consistent with these data, KDM5 genetically and physically interacts with the established stress-resistance transcription factor Foxo, and these proteins co-occupy FHRE response elements within promoters. In the absence of KDM5, Foxo recruitment to a subset of its target genes is attenuated. Significantly, this correlates with higher levels of Foxo acetylation, which is known to affect Foxo DNA binding. This leads us to propose a model in which KDM5 acts to facilitate Foxo DNA binding by recruiting HDAC4, resulting in transcriptional activation of a subset of stress resistance genes.

### Transcriptional regulation of redox states by KDM5

A total of 901 genes were up- or downregulated more than 1.5-fold in our microarray analyses of *kdm5^10424^* mutant wing imaginal discs. All genes tested as part of our validation were similarly affected in *kdm5^10424^* mutants in addition to *kdm5^10424^/kdm5^K6801^* larvae, suggesting that our expression data are robust. Interestingly, our data are significantly different to microarray analyses using wing discs from KDM5 knockdown animals recently reported, in which only 42 genes were altered more than 1.5-fold (11 upregulated and 31 downregulated) [62]. Indeed, none of the 42 genes that were affected in these KDM5 knockdown animals were altered in our analyses of *kdm5* mutant wing discs. These differences could be related the fact that we used four-fold more RNA for our transcriptome analyses, allowing us to better detect moderate changes to gene expression. Another contributing factor could be differences due to using KDM5 knockdown animals rather than genetic mutants. Whatever the basis for the disparity, our microarrays enabled us to link KDM5 to the regulation of cellular redox state for the first time.

Our observation that KDM5 affects cellular levels of oxidative stress is particularly relevant in light of evidence that dysregulated redox state contributes to the pathogenesis of several human diseases [5], [63]. Human KDM5A and KDM5B are overexpressed in many forms of cancer [3], [64]. While the role of KDM5A remains less characterized, KDM5B is specifically implicated in the survival of cancer “stem cells” [23], [24]. By promoting the survival and slow proliferation of this small subset of cells, KDM5B overexpression creates tumors that are difficult to effectively treat with standard therapies that target rapidly dividing cells. Because transformed cells have elevated levels of reactive oxygen species (reviewed in [63]), increasing the levels of KDM5A and/or KDM5B may promote cell survival. It is interesting to note that targeting KDM5-dependent demethylase activity has been proposed as a new therapeutic strategy for treating KDM5A/B-overexpressing tumors [3]. However, KDM5A was recently shown to promote breast cancer progression and metastasis in a demethylase-independent manner [65]. Taken with our data demonstrating that KDM5 acts to regulate cellular oxidative stress independently of its enzymatic activity, we suggest that approach may not yield any significant clinical improvement for these patients.

To-date, 22 mutations in the human KDM5C paralog have been found in patients with X-linked intellectual disability [29]–[32]. Increased oxidative stress has been proposed to contribute to the cognitive phenotypes of diseases including Down syndrome [34], Rett syndrome [66] and Alzheimer's disease [67]–[69]. In light of our analyses, it is possible that oxidative stress-mediated cellular damage may contribute to the intellectual impairment and other phenotypes associated with by loss of KDM5C in humans such as seizures. In the absence of KDM5C, increased oxidative damage to proteins and DNA may ultimately result in cellular dysfunction and/or death. Because KDM5C is most highly expressed in neuronal cells, other KDM5 family proteins may be unable to compensate for its loss, resulting in this cell type being vulnerable to the loss of this paralog [70]. Based on this model, we are currently examining the role of KDM5-mediated oxidative stress resistance and its relationship to learning and memory defects.

### KDM5-mediated regulation of Foxo promoter recruitment

Our data show that KDM5 regulates levels of cellular oxidative stress, at least in part through its interaction with Foxo. Indeed, levels of Foxo family proteins in human cells may therefore influence the tumorigenic and cognitive phenotypes observed as a result of KDM5 protein dysfunction. Importantly, KDM5 does not affect Foxo recruitment to all of its known target genes. This is likely to be because KDM5 is present in a subset of Foxo complexes, so only alters the acetylation of a specific pool of Foxo proteins. Consistent with this model, we find that two genes that require HDAC4 and Foxo for their activation, *InR* and *puc*, were not downregulated in *kdm5* mutants. Thus HDAC4 likely plays a more global role in Foxo acetylation than KDM5. While it remains to be determined why some Foxo targets and not others require a KDM5, the observation that Foxo requires different co-factors at distinct targets is not unprecedented. For example, Foxo requires the SWI/SNF chromatin remodeling complex at about a third of its target genes [71]. Different chromatin contexts and nuclear microenvironments may therefore require Foxo to utilize different co-factors. Promoter-specific requirements for co-factors have also been described for other transcription factors (e.g. Myc and Hsf) [72], [73]. In addition to facilitating Foxo deacetylation and activation of DNA binding capabilities, KDM5's may play additional roles in the activation of KDM5-Foxo targets. Specifically, KDM5 may use the ability to recognize specific chromatin contexts to facilitate transcriptional activation; the PHD1 of KDM5 recognizes histone H3 that is unmethylated at lysine 4 (H3K4me0) and PHD3 recognizes histone H3 that is di- and trimethylated at lysine 4 (H3K4me2/3) [15], [64].

Based on our observation that KDM5 interacts with HDAC4, we suggest that it by recruiting this enzyme that KDM5 affects Foxo acetylation (Figure 7). Alternatively, KDM5 could affect the deacetylase activity of HDAC4 within the KDM5-Foxo complexes to regulate levels of Foxo acetylation, since KDM5 can inhibit HDAC1 [18], [19]. Even though KDM5 and HDAC4 interact, it is also possible that KDM5 affects Foxo acetylation through an HDAC4-independent mechanism. For example, Sir2 has also been shown to deacetylate Foxo [74], [75], and the relative functions of HDAC4 and Sir2 remain unclear. KDM5 could therefore influence Foxo acetylation through Sir2. Foxo acetylation is also positively regulated by the lysine acetyltransferase CBP/p300, thus it is also formally possible that KDM5 acts by negatively regulating CBP/p300 activity.

### KDM5-dependent and independent Foxo complexes

The KDM5-Foxo complex is also likely to be important for cellular processes in addition to oxidative stress resistance. For example, based on the genetic interaction we observed between *kdm5* and *foxo* in the eye, KDM5 and Foxo may act together to regulate cell size and number in some circumstances. In addition, the KDM5-Foxo complex may also function to influence aging. Levels of Foxo influence lifespan in a number of species: reducing Foxo shortens lifespan while increasing Foxo extends it [76]. In a similar manner to *foxo* mutant flies, the hypomorphic *kdm5* allele combination of *kdm5^10424^*/*kdm5^k6801^* dramatically shortens, and adult specific KDM5 knockdown slightly shortens, lifespan. This is likely to be a conserved function of KDM5 family proteins, as loss of KDM5 (Rbr-2) in *c. elegans* shortens lifespan and its overexpression extends lifespan [77], [78]. It will therefore be important to determine whether adult-specific overexpression of KDM5 extends lifespan in *Drosophila*, and whether this is dependent on levels of Foxo. Because there is a correlation between redox state and aging [79], effects of the KDM5/Foxo complex on lifespan and aging could be mediated through their regulation of oxidative stress resistance genes.

Although *kdm5* and *foxo* mutants phenocopy each other in some regards, both KDM5 and Foxo also have roles outside this complex. This is based on the observations that not all Foxo-regulated oxidative stress resistance genes are affected by loss of KDM5 and that while *kdm5* is an essential gene, *foxo* null mutants are homozygous viable [22], [80]. Based on our finding that KDM5 affects Foxo acetylation and promoter recruitment, it is tempting to speculate that one of the reasons that KDM5 is essential for viability is that it acts through a similar mechanism to influence the activity of other transcription factors. Many transcription factors are acetylated, including Myc, which we have shown interacts with KDM5, in addition to p53, GATA and STAT family proteins [59], [81]–[83]. KDM5 could therefore interact with these factors to impact a broad range of essential cellular functions.

## Materials and Methods

### *Drosophila* stocks

All fly stocks were maintained at 25°C on standard medium, 60% humidity, and a 12 hour light/dark cycle. *kdm5* mutant alleles *kdm5^10424^*, *kdm5^K06801^*, UAS-KDM5^RNAi^ (TRIP line 35706), UAS-Foxo, Actin-Gal4, tubulin-Gal80^ts^, FRT40A, hs-FLP^122^, GMR-Gal4, *foxo^Δ94^* (maintained as a heterozygous stock over TM6B) and HDAC4^KG09091^ are publically available and were obtained from the Bloomington stock center. *foxo^21^* was obtained from Marc Tatar [84] and was maintained as a homozygous stock. UAS-KDM5^RNAi^ was generated by cloning an inverted repeat of KDM5 into the *BamHI* site of pMF3 using the primers gcggatccgtacatgcagcgtcagcggcaac (*BamHI* site underlined) and gcgaattccgcattattgcctccagtagctg (*EcoRI* site used to join inverted repeats underlined). Control UAS-GFP^RNAi^ transgenic flies were generated by cloning an inverted repeat as a *BamHI* fragment using the primers gcggatccctggaaaactacctgttccatg (*BamHI* site underlined) and gcgaattcgttcatccatgccatgtgtaatc (*EcoRI* site that joins two repeats underlined).

To generate *kdm5* mutant clones, we crossed hs-FLP^122^; FRT40A ubi-GFP virgins to +; *kdm5^K6801^* FRT40/CyO males. Progeny were heat shocked for 45 min at 48 hours AED, and larvae were dissected at wandering 3^rd^ instar stage. As a control, hs-FLP^122^; FRT40A ubi-GFP virgins were crossed to FRT40/CyO males. Clone size was determined by quantifying the total area of dark GFP positive (twin spot): GFP negative (control *wt* or *kdm5* mutant cells) using Image J.

The HA-tagged KDM5 genomic rescue transgene was generated by inserting the coding sequence for three HA tags (YPYDVPDYA) at the 3′end of the *kdm5* open reading frame after removal of the endogenous stop codon. This *kdm5-HA* open reading frame fragment was then cloned downstream of the *kdm5* promoter in the pCasper 4 vector as described for our non-tagged genomic rescue transgene [15]. *kdm5*-HA transgenic flies were crossed into a *kdm5* (*kdm5^10424^* or *kdm5^K06801^*) mutant background. Insertions on the 3^rd^ chromosome and X chromosome were used and behaved indistinguishably. Transgenic flies were generated by “thebestgene.com”.

### Antibodies and Westerns

The KDM5 antibody has been described previously [13], [85], anti-Foxo antibodies were obtained from Marc Tatar (Brown University; used for ) and from Cosmo Bio USA (used for Figure 6) [86], anti-H3K4me3 and anti-histone H3 were from Active Motif, γ-tubulin from Cell Signaling, anti-pan acetylated H3 from Upstate and anti-HA from Invitrogen. To detect *Drosophila* HDAC4, we used an antibody raised to human HDAC4, 5 and 9 from Novus. In *Drosophila*, our Western analyses show that this antibody recognizes a single band of the predicted molecular weight for HDAC4 (125 kDa) and that this co-migrates with a transfected FLAG-tagged form of HDAC4 (Figure S10). We therefore conclude that this antibody is specific to HDAC4 in flies. Western analysis was carried out using PVDF and standard protocols, infrared conjugated secondary antibodies (LiCOR) and Odyssey scanner and software. Signal intensities were quantitated using LiCOR software.

### *In vitro* and *in vivo* binding assays

*In vitro* GST-fusion protein binding assays were performed as described previously [87]. For immunoprecipitations of Foxo, S2 cell nuclei with or without 20 mM paraquat treatment were isolated and fractionated using the Nuclear Extract Kit (Active Motif). Nuclear extract was dilute to 1 ml in cold immunoprecipitation buffer (20 mM HEPES, PH 7.5, 50 mM KCl, 0.05% Triton X-100, 2.5 mM EDTA, 5 mM DTT, 5% glycerol, 10 mg/ml protease inhibitor cocktail (Fisher). Extracts were pre-cleared for 30 minutes with 30 µl Protein G Sepharose (Invitrogen) in a total volume of 500 µl. After centrifugation, the supernatant was used to carry out immunoprecipitation by incubation with anti-Foxo or anti-KDM5. For immunopreciptations of HDAC4, total cell lysates were used and processed as described above for Foxo.

### RT-PCR

Whole larva or dissected tissue were added to TRIZOL (Invitrogen) to extract RNA followed by cleanup using DNA-free (Ambion). 500 ng of total RNA was reverse transcribed at 42°C for 30 minutes using Verso cDNA kit (Thermo Scientific) with oligo (dt) primer to generate cDNA. Quantitative real-time PCR (qRT-PCR) analysis was performed using SYBR Green Master Mix (Thermo scientific). qRT-PCR reactions were performed in triplicate in total volumes of 10 µl containing Fast SYBR Green Master Mix, 0.25 µl of each gene-specific primer, 0.5 µl of first strand cDNA template, and nuclease free water. All qRT-PCR reactions were performed using Applied Biosystems StepOnePlus real time PCR system with the following conditions: 95°C for 15 min followed by 40 cycles of denaturation at 95°C for 15 s, annealing at 60°C for 30 s and extension at 72 for 30 s. Data analysis was performed using StepOne software (Applied Biosystems). Fold change was calculated using s-DDCT method [88]. At least three biological replicates were used for each primer set. Primer sequences are provided in Table S2. Levels of gene expression in each sample were normalized to the expression of the housekeeping gene *rp49*.

### Paraquat and DDT treatment

Larvae were treated with 20 mM paraquat for six hours in 5% sucrose (or sucrose alone as a control). Adults were treated with 20 mM paraquat by placing them in a vial with a paraquat-soaked (or sucrose alone) piece of whatman paper. Adults were treated with the insecticide DDT according to a previously published protocol [89].

### Gene array processing and statistical analysis

Total RNA was prepared from three separate samples of 60 dissected *w^1118^* and *kdm5^10424^* wing imaginal discs (96 hr AED) using Trizol, followed by on-column digestion of DNA using the RNeasy Mini Kit (Qiagen). RNA quantity and quality were assessed with a Beckman Coulter DU 640 spectrophotometer and Agilent 2100 Bioanalyzer, following the manufacturer's protocols. Biotinylated, single-stranded cDNA was prepared from 100 ng total intact RNA. cDNA was hybridized to *Drosophila* Genome 2.0 GeneChips (Affymetrix). After hybridization, GeneChips were scanned using a GeneChip Scanner 3000 (Affymetrix). CEL files were generated from DAT files using GCOS software (Affymetrix). Data were processed with Expression Console (Affymetrix) using the PLIER algorithm and Array Assist Lite software was used to generate GC-RMA files (log2 transformed) for each chip. All procedures were performed in three biological replicates at the Genome Center of the Albert Einstein College of Medicine. Fold change was calculated for *kdm5* ^10424^ relative to wildtype (*wt*; *w^1118^*). Statistical significance (*p* value) was calculated by Student's t-test, based on the results of three arrays from *wt* and *kdm5* ^10424^. Genes that changed by less than 1.5-fold and had a *p* value more than 0.05 were removed from subsequent analysis. The normalized RMA values from these genes (affected ≥1.5 fold, *p*<0.05) were used to perform a hierarchical cluster analysis and to construct a heat map using the Gene Cluster 3.0 and tree view software [90]. Cluster analysis groups together genes with comparable patterns of expression by employing euclidean distance metric and the Centroid linkage method. The false discovery rate (Q value) was calculated for each *p*-value using R [91], [92]. The accession number for microarray data is GSZ53881.

### Functional interpretation of microarray data

Degree of enrichment for cellular component, biological processes and molecular functions was assessed using the Gene ontology (GO) program DAVID and Easy GO [93]–[95]. Hierarchical cluster analysis was to construct a heat map using Gene Cluster 3.0 and Java tree view software [90].

### *lacZ* mutation analyses and protein oxidation assay

To determine *lacZ* mutation frequency in female larvae, #9lacZ or *kdm5^10424^*, #9lacZ homozygous larvae were selected. Both the #9lacZ transgene and the *kdm5^10424^*, #9lacZ recombinant chromosome were crossed into a *w^1118^* genetic background. The *lacZ* transgenes, procedures for DNA extraction and mutation frequency determination have been previously described [51]. Total levels of oxidized protein was determined using “oxyblot” from Millipore according to manufacturer specifications.

### Chromatin immunoprecipitation

ChIP was performed using the anterior half of 3^rd^ instar larvae essentially as previously described [96]. Cross-linking was performed for 25 min using 1.8% formaldehyde during tissue homogenization. Chromatin extracts were sonicated using a Bioruptor (Diagenode) for 25 min (settings 30 sec on, 30 sec off, high power) to give rise to sheared chromatin with an average length of 200 to 800 bp. Immunoprecipitations were performed using 2–4 µg of anti-Foxo, anti-KDM5, anti-H3K4me3, anti-H3Ac or anti-HA. For ChIP-qPCR, triplicates from two independent biological replicates were analyzed following the ΔCt method. Data are expressed as the percentage of input chromatin precipitated for each region examined. Table S2 lists the sequences of primers used in these experiments.

### Statistical analyses

The statistical significance of gene expression and ChIP binding were determined using a student's t-test (Microsoft excel). Statistical significance of lifespan data were determined with Log-rank (Mantel-cox) test using Prism (GraphPad software). Eye size of GMR>Foxo and other genotypes was determined using ImageJ and statistical significance determined using a student's t-test. R^2^ correlation coefficients for microarray biological replicates were generated using ArrayStar Scatter Plot (DNAStar) and are shown in Figure S1.

## Supporting Information

Figure S1 Microarray analyses of *kdm5* mutant wing imaginal discs. (A–C) Correlation graphs and R^2^ values for the three repeats of wildtype imaginal disc microarray data. (D–F) Correlation graphs and R2 values of the three repeats from *kdm5^10424^* mutant wing imaginal discs. All R^2^ values are highly significant, demonstrating the reproducibility of our data. (G) Distribution of the number of genes affected in *kdm5* mutant wing discs > = 1.5-fold, 1.5–2-fold, 2–5 fold and 5–10-fold. (H) Comparison of changes to gene expression observed from microarray analyses (blue) and real-time PCR carried out in triplicate (red). Samples are normalized to expression of *rp49* within each sample and shown relative to control (*w^1118^*) animals.
(TIF)

Figure S2 Oxidation-reduction gene expression changes in *kdm5^10424^* mutants. (A) Heat map based on microarray data showing the expression of genes significantly affected in *kdm5^10424^* wing discs in the gene ontology “stress response” category. (B) Heat map based on microarray data showing levels of gene expression of genes in the general gene ontology category oxidation-reduction. (C) Molecular function analyses of genes affected in *kdm5^10424^* mutant wing imaginal discs as determined by the gene ontology (GO) program, Easy GO.
(JPG)

Figure S3 Survival of *kdm5* mutant adults in non-stressed and oxidative stress conditions. (A) Survival of control (*w^1118^*) and *kdm5^K6801/10424^* adult males fed 20 mM paraquat in 5% sucrose. Average of three experiments shown. Survival curves are significantly different from one another (*p*<0.01). (B) Lifespan of control (*w^1118^*) and *kdm5^K6801/10424^* female flies. Median lifespans were 9 and 71 days for *kdm5^K6801/10424^* and control, respectively. These lifespans are significantly different from one another (*p*<<0.01). (C) Survival of control (*w^1118^*) and *kdm5^K6801/10424^* adult males in non-stressed conditions. Survival curves are significantly different from one another (*p*<<0.01). Median lifespans were 8 and 65 days for *w^1118^* and *kdm5^K6801/10424^*, respectively.
(TIF)

Figure S4 KDM5 knockdown phenotypes in non-stressed and oxidative stress conditions. (A) Western blot showing levels of KDM5 and the loading control histone H3 in Act^TS^>GFP^RNAi^ and Act^TS^>KDM5^RNAi^ adult female heads after five days at 25°C. (B) Lifespan of control tubulin-Gal4/+; Actin-Gal4/UAS-GFP^RNAi^ (Act^TS^>GFP^RNAi^), and tubulin-Gal4/+; Actin-Gal4/UAS-KDM5^RNAi^ (Act^TS^>KDM5^RNAi^) female flies at 25°C. Median lifespans were 67 and 72 days for Act^TS^>KDM5^RNAi^ and control, respectively, which are significantly different from one another (*p*<0.05). The cross to generate flies was carried out at 18°C then adults of the correct genotype were transferred to 25°C. (C) Survival curve of control (Act^TS^>GFP^RNAi^) and Act^TS^>KDM5^RNAi^ female adults fed 20 mM paraquat in 5% sucrose at 25°C. Error bars show standard error. The two survival curves are significantly different from one another (*p*<0.05). (D) Western blot of KDM5 levels and the loading control γ-tubulin from whole adults at 18°C. Genotypes are Act^TS^>GFP^RNAi^ and Act^TS^>KDM5^RNAi^. (E) Survival curve of control (Act^TS^>GFP^RNAi^) and Act^TS^>KDM5^RNAi^ female adults fed 20 mM paraquat in 5% sucrose at 18°C. The two survival curves are not statistically significantly different from one another. (F) Survival curve of control (Act^TS^>GFP^RNAi^) and Act^TS^>KDM5^RNAi^ female adults fed the insecticide DDT. The survival curves are significantly different from one another (*p*<0.05).
(TIF)

Figure S5 *foxo* mutant paraquat sensitivity, quantifying the KDM5-Foxo interaction and assessing gene expression in response to paraquat. (A) Survival curve of control (*w^1118^*) and *foxo^21^* homozygous mutant adults fed 20 mM paraquat. The two survival curves are significantly different from one another (*p*<0.01). (B) Quantification from three experiments of the *in vitro* interaction observed between GST-Foxo and *in vitro* transcribed/translated KDM5. ** *p*<0.01. (C) Quantification of the *in vivo* interaction observed between Foxo and KDM5 from three independent experiments. ** *p*<0.01. (D) Real-time PCR analyses of wildtype (*w^1118^*) 3^rd^ instar larvae placed in 20 mM paraquat/5% sucrose for six hours. mRNA levels are shown normalized to larvae placed in 5% sucrose for 6 hours. * indicates *p*<0.05.
(TIF)

Figure S6 Chromatin analyses of KDM5-Foxo target genes. (A) Schematic of the promoters of *InR*, *puc*, *4E-BP*, *l(2)efl*, *CG5316*, *spirit*, *Prx2540-2* and *CG10211* showing the position of the Foxo binding sites (FHREs; black boxes). Primers surrounding these sites were used for ChIP analyses shown in B. (B) Foxo ChIP (using the antibody obtained from the Tatar lab) to the FHRE region of *InR*, *puc*, *4E-BP*, *l(2)efl*, *CG5316*, *spirit*, *Prx2540-2* and *CG10211* in wildtype (*w^1118^*) and *kdm5^10424/K6801^* mutant larvae. IgG is included as an additional control for non-specific binding in wildtype and *kdm5^10424/K6801^* mutant larvae. (C) Anti-acetylated histone H3 ChIP analyses surrounding the Foxo binding site in wildtype (*w^1118^*) and *kdm5^10424/K6801^* mutant larvae. * *p*<0.05.
(TIF)

Figure S7 KDM5 and Foxo do not bind to specific non-promoter regions of oxidative stress resistance genes. (A) Schematic of *InR*, *puc*, *4E-BP*, *l(2)efl*, *CG5316*, *spirit*, *Prx2540-2* and *CG10211* genes showing the position of ChIP primers. (B) Anti-Foxo ChIP (using Antibody from Cosmo Bio) showing no difference in binding in wildtype and *foxo^21^* mutant larvae. (C) Anti-KDM5 ChIP to non-promoter regions showing no KDM5 enrichment. ns = not statistically different.
(TIF)

Figure S8 A HA-tagged genomic-rescue KDM5 transgene directly binds to oxidative stress resistance genes. (A) Western analyses of wildtype (*w^1118^*; left lane) 3^rd^ instar larvae in addition to *kdm5^K6801^* mutants carrying two copies of the gKDM5:HA transgene (middle lane) or without any transgene (right land). Levels of KDM5, HA and control histone H3 are shown. Eight imaginal discs were loaded per lane. * indicates non-specific band. (B) Comparison of the ability of untagged gKDM5 and gKDM5:HA to rescue *kdm5^K6801^* mutants to viability. gKDM5 and gKDM5:HA rescue equally well. Results expressed as percent of expected progeny from the intercross of *kdm5^K6801^*/CyO; gKDM5 (or gKDM5:HA). At least 100 progeny were scored. ns = not significant. (C–G) ChIP analyses using anti-HA compared to IgG control in 3^rd^ instar larvae. Promoters examined were *4E-BP*, *l(2)efl*, *CG5316*, and *Prx2540-2*. *InR* is a Foxo-regulated gene that is not a KDM5 target so serves as a negative control. * indicates *p*<0.05.
(TIF)

Figure S9 Quantitation of protein levels. (A–D) Quantitation of levels of HDAC4, Foxo, Acetylated Foxo and γ-tubulin (control) in wildtype (*w^1118^*) and *kdm5^K6801^* homozygous larvae from three independent experiments as determined using LiCOR software. Protein levels are expressed relative to levels observed in wildtype. *p<0.05. (E, F) Quantitation of levels of HDAC4 and KDM5 in samples used for co-immunoprecipitation analyses. Protein levels are shown relative to levels observed in 10% input lane. * *p*<0.01.
(TIF)

Figure S10 Specificity of HDAC4 antibody. Western blot analyses of S2 cell extract mock transfected (−) or transfected with a HDAC4:FLAG construct using anti-FLAG (left), an antibody that recognizes human HDAC4, 5, and 9 (middle) and a merge of the two channels (right). The co-migration of endogenous HDAC4 and FLAG-tagged HDAC4 shows that this antibody is specific to HDAC4 in *Drosophila*.
(TIF)

Table S1 Adult survival of kdm5 alleles.
(PDF)

Table S2 Primers used for real-time PCR and ChIP.
(PDF)
