# Supplementary figures and images for: KDM5 Interacts with Foxo to Modulate Cellular Levels of Oxidative Stress
Source: PLoS Genet. 2014 Oct 16;10(10):e1004676. doi: 10.1371/journal.pgen.1004676 (PMC4199495; doi:10.1371/journal.pgen.1004676)

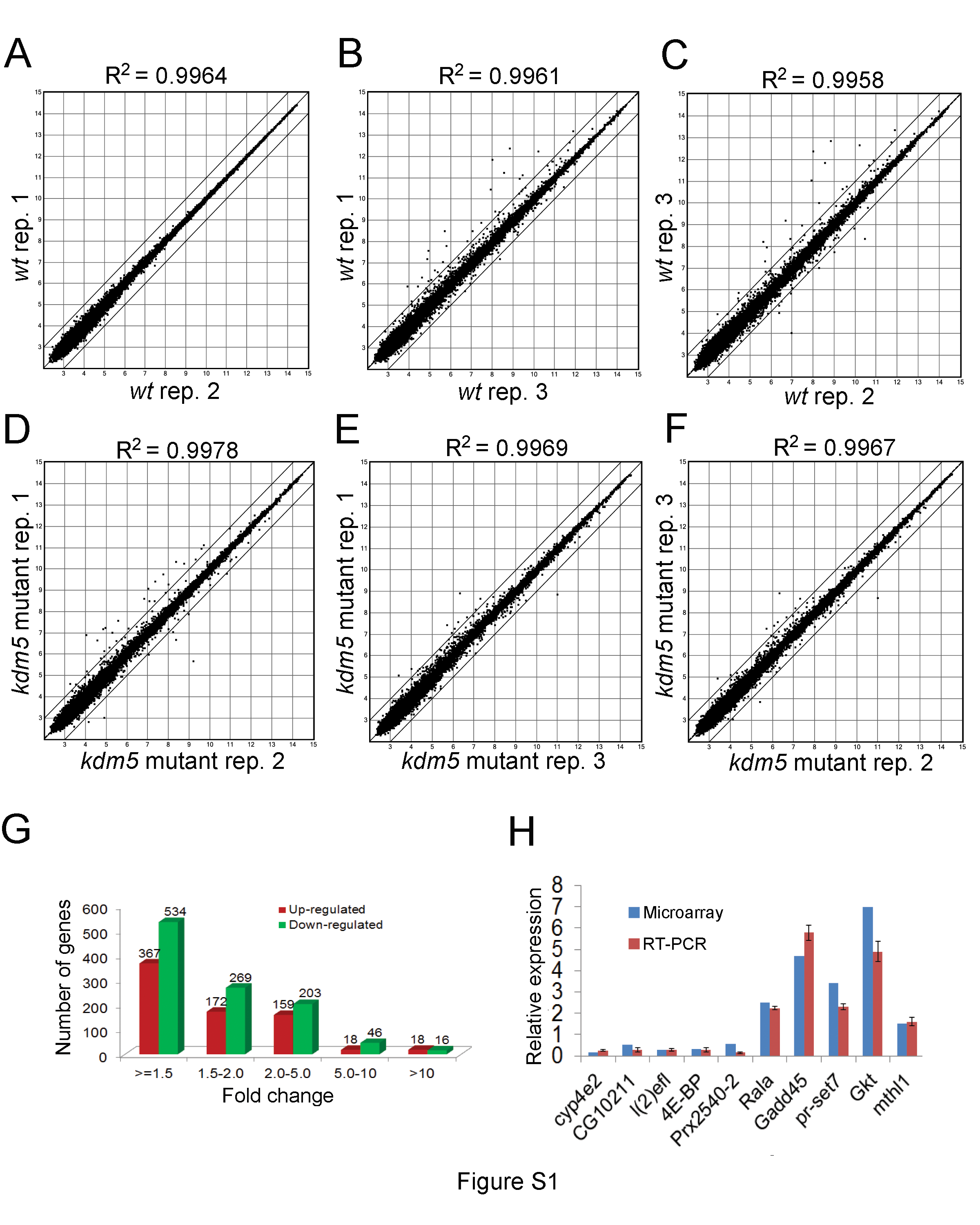

Supplement: Figure S1 — Microarray analyses of kdm5 mutant wing imaginal discs. (A–C) Correlation graphs and R2 values for the three repeats of wildtype imaginal disc microarray data. (D–F) Correlation graphs and R2 values of the three repeats from kdm510424 mutant wing imaginal discs. All R2 values are highly significant, demonstrating the reproducibility of our data. (G) Distribution of the number of genes affected in kdm5 mutant wing discs > = 1.5-fold, 1.5–2-fold, 2–5 fold and 5–10-fold. (H) Comparison of changes to gene expression observed from microarray analyses (blue) and real-time PCR carried out in triplicate (red). Samples are normalized to expression of rp49 within each sample and shown relative to control (w1118) animals. (TIF) [file pgen.1004676.s001.tif]

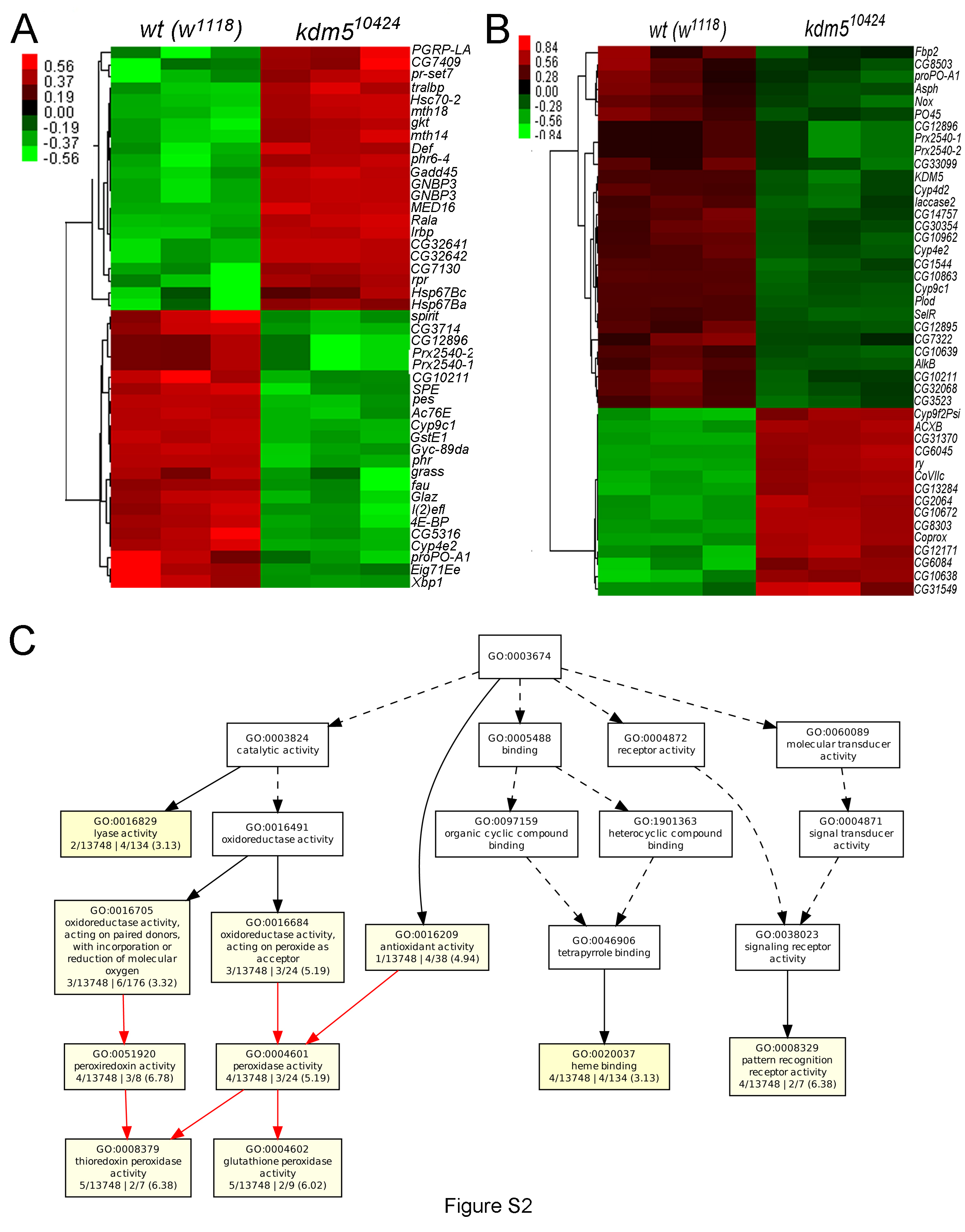

Supplement: Figure S2 — Oxidation-reduction gene expression changes in kdm510424 mutants. (A) Heat map based on microarray data showing the expression of genes significantly affected in kdm510424 wing discs in the gene ontology “stress response” category. (B) Heat map based on microarray data showing levels of gene expression of genes in the general gene ontology category oxidation-reduction. (C) Molecular function analyses of genes affected in kdm510424 mutant wing imaginal discs as determined by the gene ontology (GO) program, Easy GO. (JPG) [file pgen.1004676.s002.jpg]

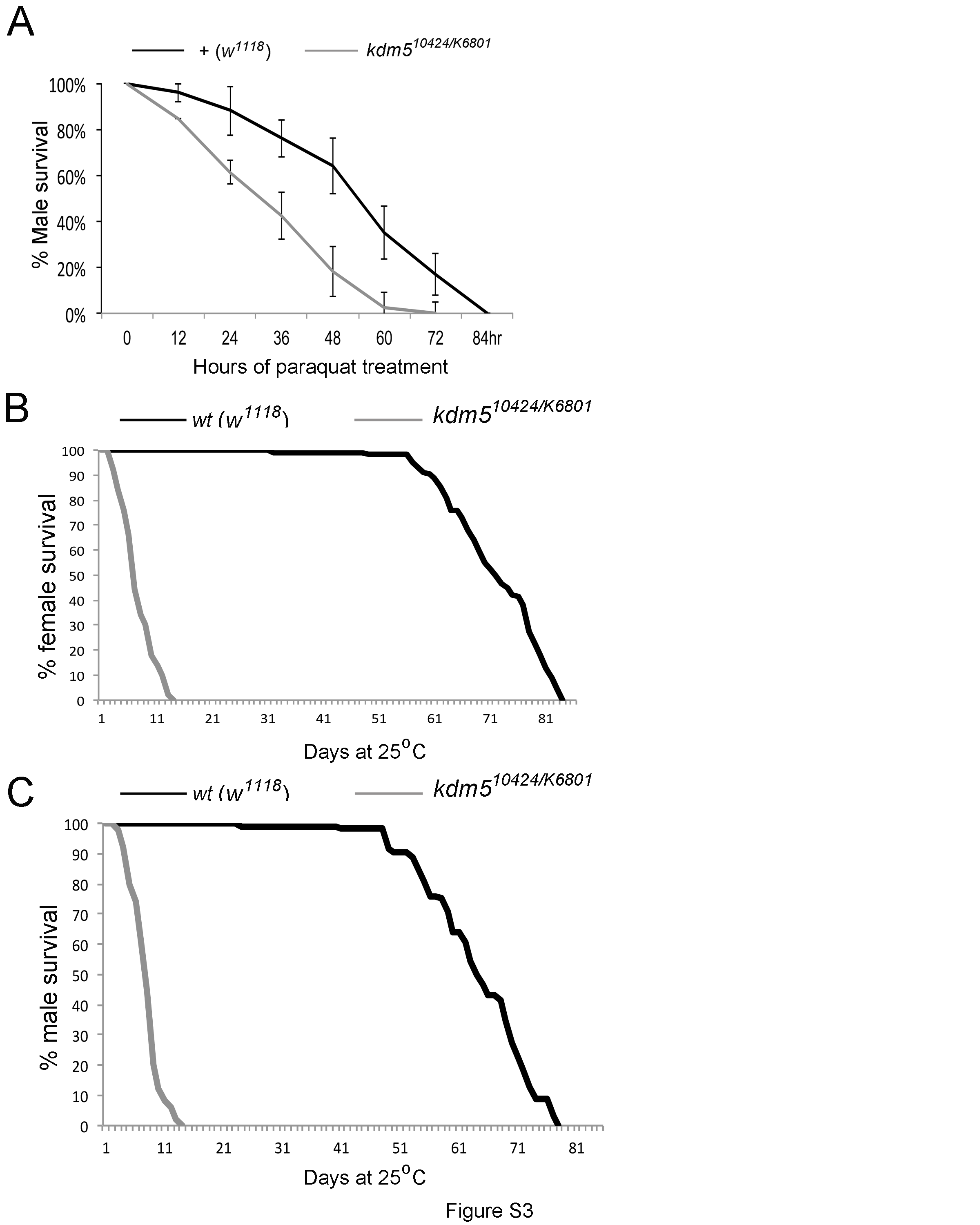

Supplement: Figure S3 — Survival of kdm5 mutant adults in non-stressed and oxidative stress conditions. (A) Survival of control (w1118) and kdm5K6801/10424 adult males fed 20 mM paraquat in 5% sucrose. Average of three experiments shown. Survival curves are significantly different from one another (p<0.01). (B) Lifespan of control (w1118) and kdm5K6801/10424 female flies. Median lifespans were 9 and 71 days for kdm5K6801/10424 and control, respectively. These lifespans are significantly different from one another (p<<0.01). (C) Survival of control (w1118) and kdm5K6801/10424 adult males in non-stressed conditions. Survival curves are significantly different from one another (p<<0.01). Median lifespans were 8 and 65 days for w1118 and kdm5K6801/10424, respectively. (TIF) [file pgen.1004676.s003.tif]

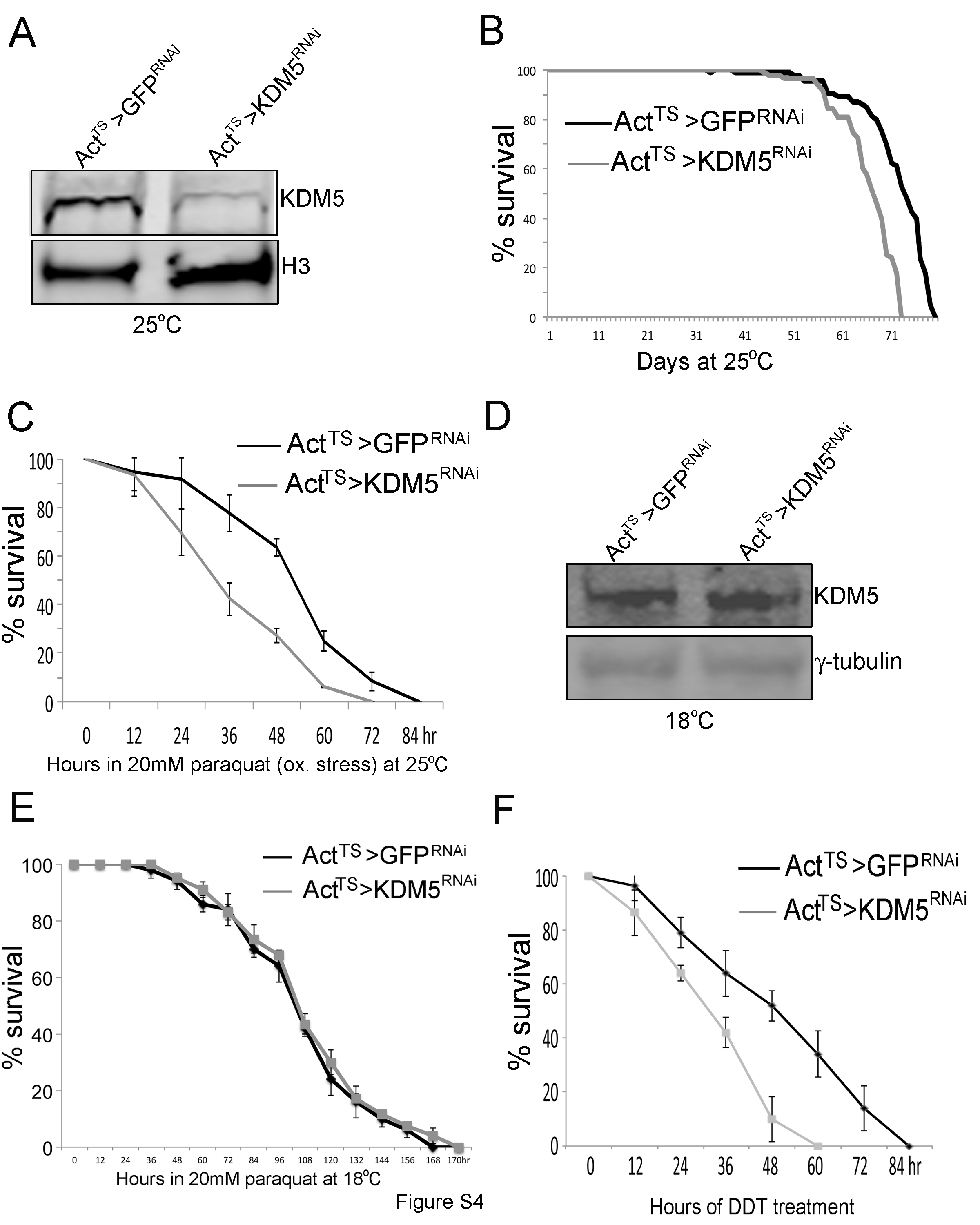

Supplement: Figure S4 — KDM5 knockdown phenotypes in non-stressed and oxidative stress conditions. (A) Western blot showing levels of KDM5 and the loading control histone H3 in ActTS>GFPRNAi and ActTS>KDM5RNAi adult female heads after five days at 25°C. (B) Lifespan of control tubulin-Gal4/+; Actin-Gal4/UAS-GFPRNAi (ActTS>GFPRNAi), and tubulin-Gal4/+; Actin-Gal4/UAS-KDM5RNAi (ActTS>KDM5RNAi) female flies at 25°C. Median lifespans were 67 and 72 days for ActTS>KDM5RNAi and control, respectively, which are significantly different from one another (p<0.05). The cross to generate flies was carried out at 18°C then adults of the correct genotype were transferred to 25°C. (C) Survival curve of control (ActTS>GFPRNAi) and ActTS>KDM5RNAi female adults fed 20 mM paraquat in 5% sucrose at 25°C. Error bars show standard error. The two survival curves are significantly different from one another (p<0.05). (D) Western blot of KDM5 levels and the loading control γ-tubulin from whole adults at 18°C. Genotypes are ActTS>GFPRNAi and ActTS>KDM5RNAi. (E) Survival curve of control (ActTS>GFPRNAi) and ActTS>KDM5RNAi female adults fed 20 mM paraquat in 5% sucrose at 18°C. The two survival curves are not statistically significantly different from one another. (F) Survival curve of control (ActTS>GFPRNAi) and ActTS>KDM5RNAi female adults fed the insecticide DDT. The survival curves are significantly different from one another (p<0.05). (TIF) [file pgen.1004676.s004.tif]

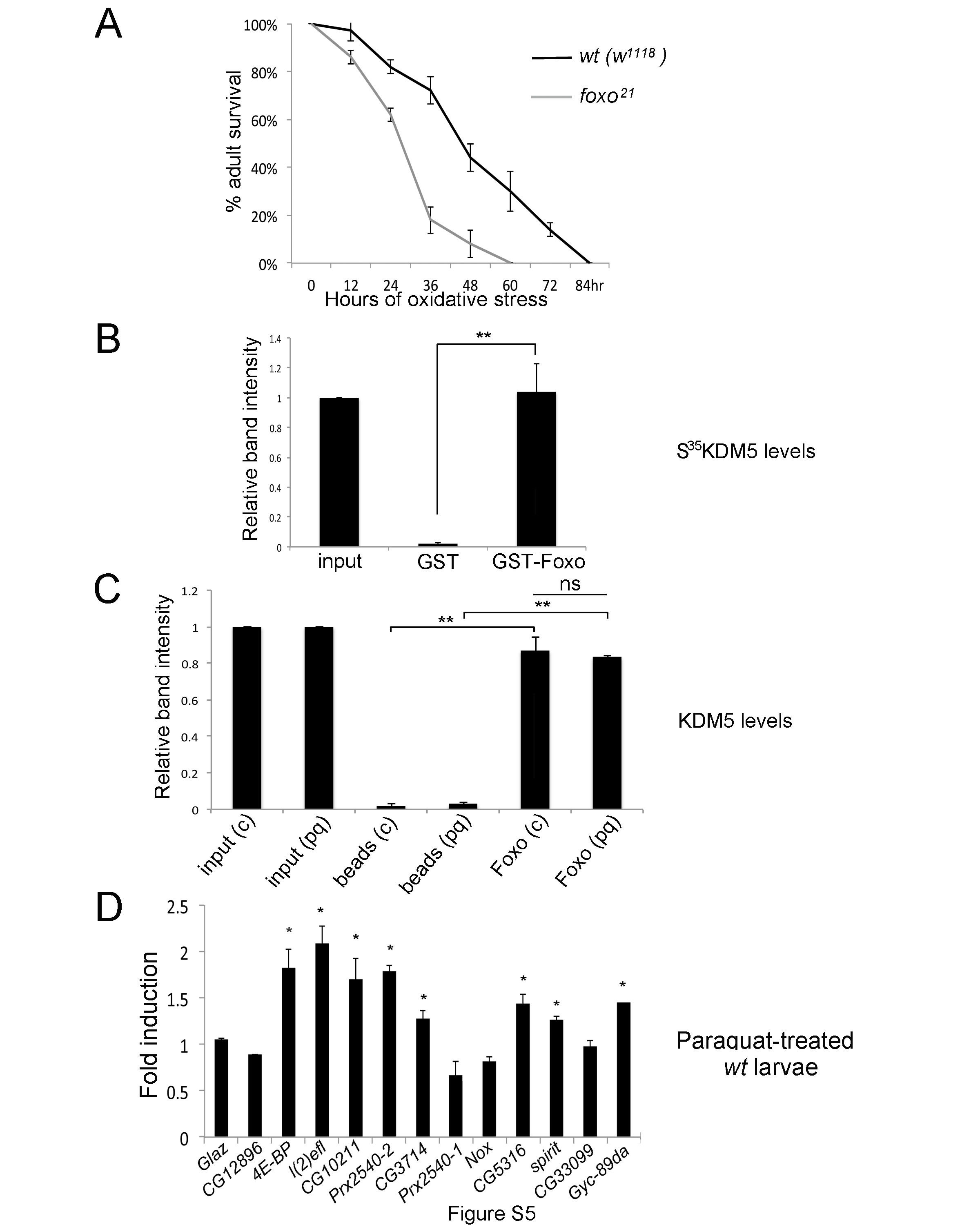

Supplement: Figure S5 — foxo mutant paraquat sensitivity, quantifying the KDM5-Foxo interaction and assessing gene expression in response to paraquat. (A) Survival curve of control (w1118) and foxo21 homozygous mutant adults fed 20 mM paraquat. The two survival curves are significantly different from one another (p<0.01). (B) Quantification from three experiments of the in vitro interaction observed between GST-Foxo and in vitro transcribed/translated KDM5. ** p<0.01. (C) Quantification of the in vivo interaction observed between Foxo and KDM5 from three independent experiments. ** p<0.01. (D) Real-time PCR analyses of wildtype (w1118) 3rd instar larvae placed in 20 mM paraquat/5% sucrose for six hours. mRNA levels are shown normalized to larvae placed in 5% sucrose for 6 hours. * indicates p<0.05. (TIF) [file pgen.1004676.s005.tif]

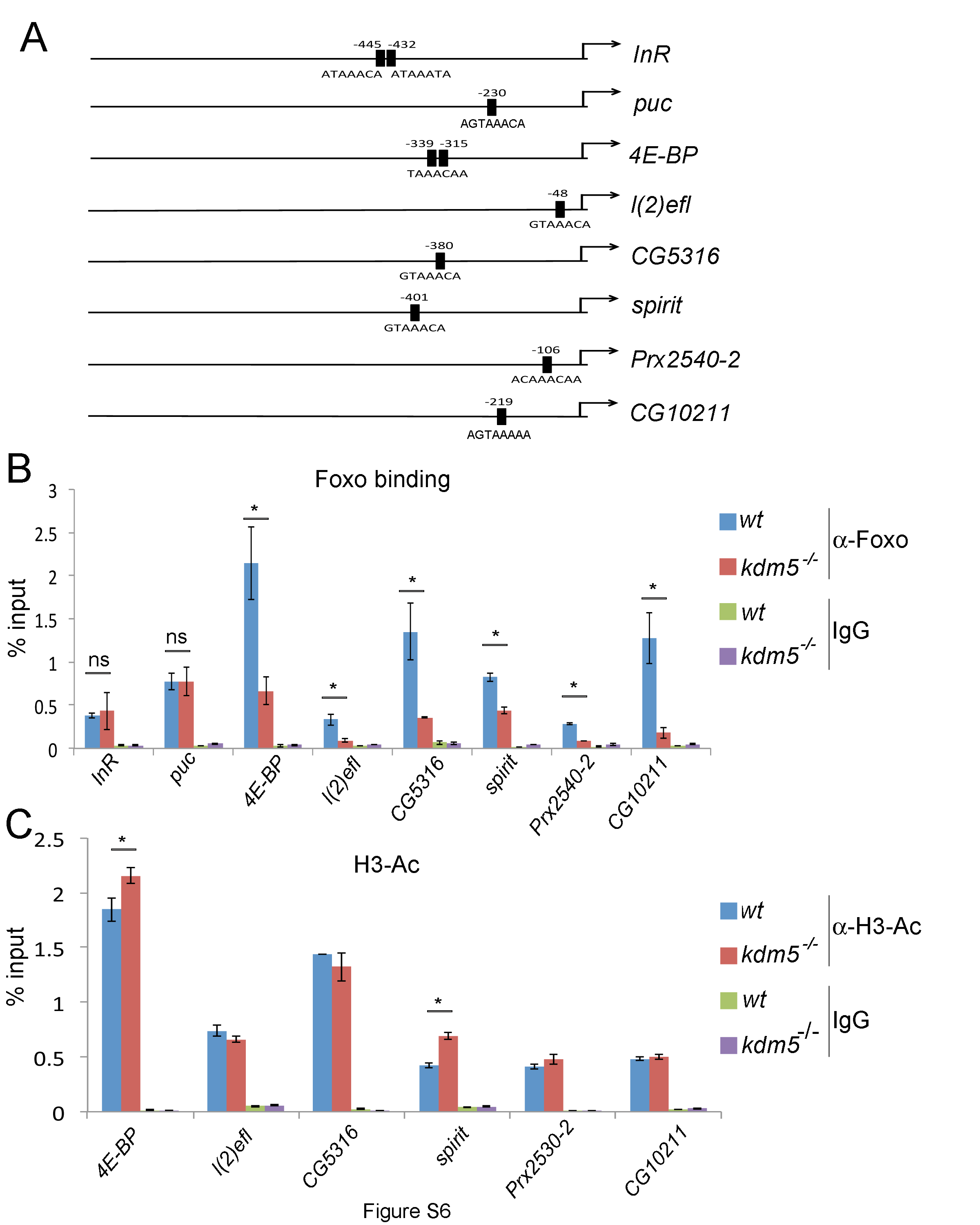

Supplement: Figure S6 — Chromatin analyses of KDM5-Foxo target genes. (A) Schematic of the promoters of InR, puc, 4E-BP, l(2)efl, CG5316, spirit, Prx2540-2 and CG10211 showing the position of the Foxo binding sites (FHREs; black boxes). Primers surrounding these sites were used for ChIP analyses shown in B. (B) Foxo ChIP (using the antibody obtained from the Tatar lab) to the FHRE region of InR, puc, 4E-BP, l(2)efl, CG5316, spirit, Prx2540-2 and CG10211 in wildtype (w1118) and kdm510424/K6801 mutant larvae. IgG is included as an additional control for non-specific binding in wildtype and kdm510424/K6801 mutant larvae. (C) Anti-acetylated histone H3 ChIP analyses surrounding the Foxo binding site in wildtype (w1118) and kdm510424/K6801 mutant larvae. * p<0.05. (TIF) [file pgen.1004676.s006.tif]

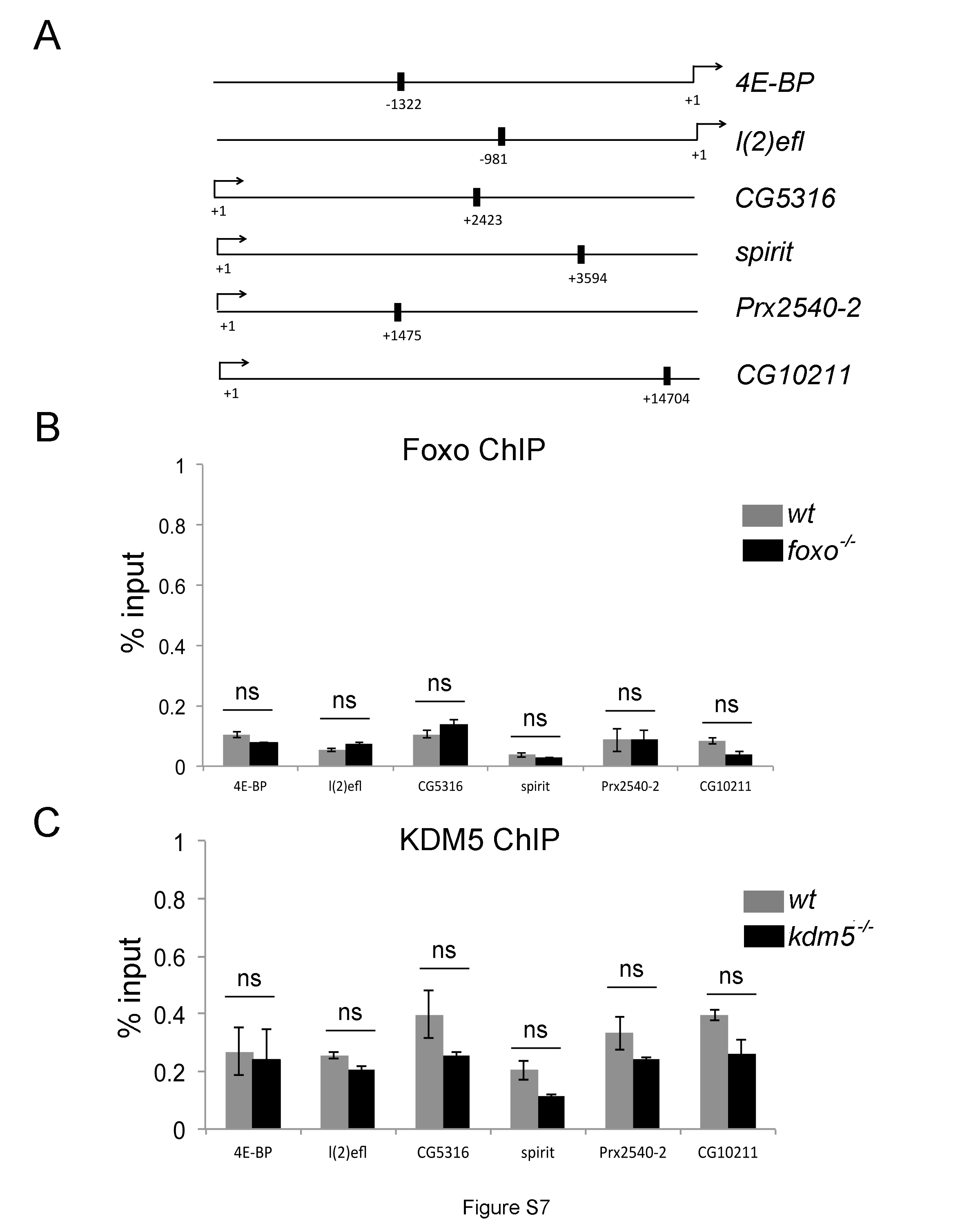

Supplement: Figure S7 — KDM5 and Foxo do not bind to specific non-promoter regions of oxidative stress resistance genes. (A) Schematic of InR, puc, 4E-BP, l(2)efl, CG5316, spirit, Prx2540-2 and CG10211 genes showing the position of ChIP primers. (B) Anti-Foxo ChIP (using Antibody from Cosmo Bio) showing no difference in binding in wildtype and foxo21 mutant larvae. (C) Anti-KDM5 ChIP to non-promoter regions showing no KDM5 enrichment. ns = not statistically different. (TIF) [file pgen.1004676.s007.tif]

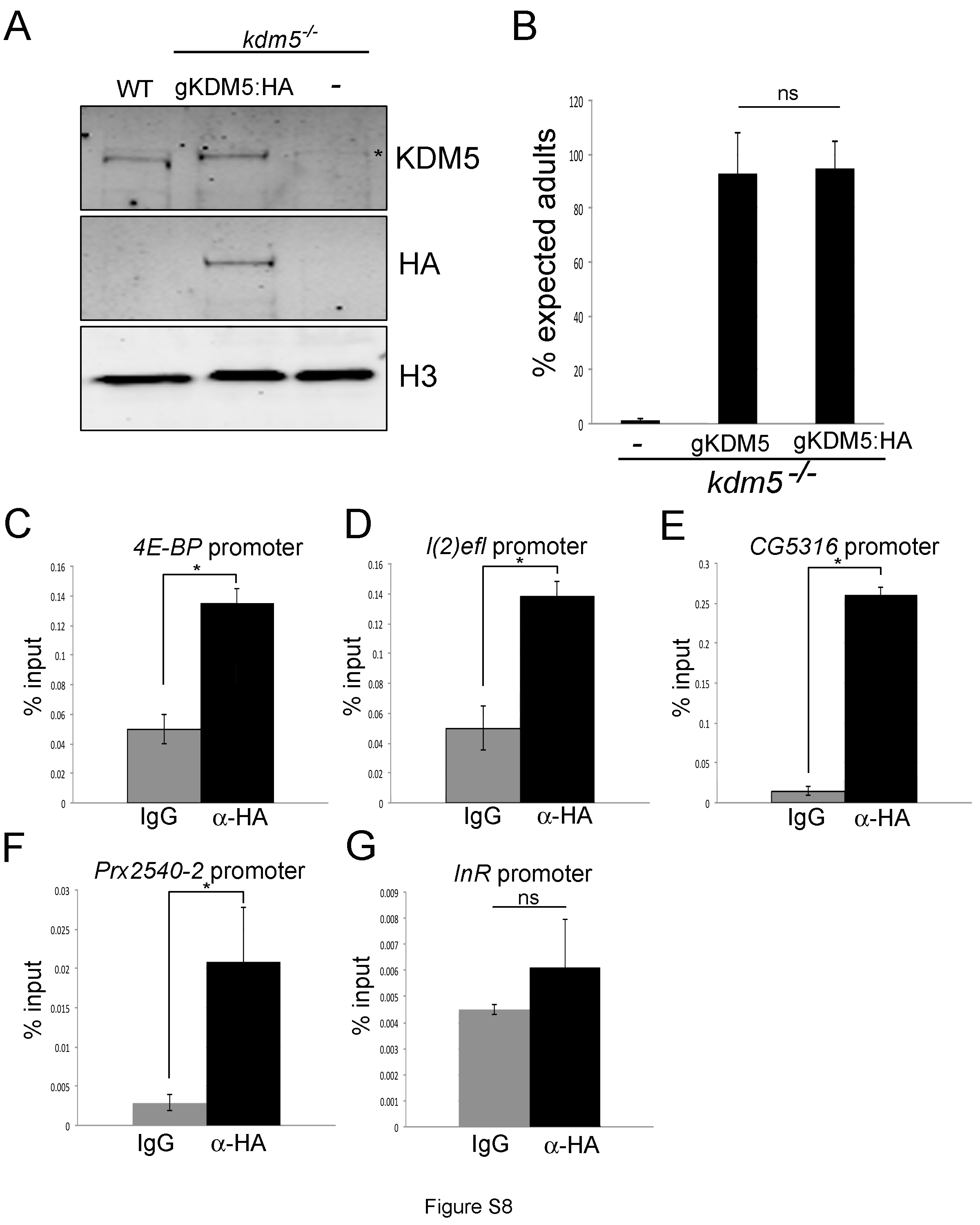

Supplement: Figure S8 — A HA-tagged genomic-rescue KDM5 transgene directly binds to oxidative stress resistance genes. (A) Western analyses of wildtype (w1118; left lane) 3rd instar larvae in addition to kdm5K6801 mutants carrying two copies of the gKDM5:HA transgene (middle lane) or without any transgene (right land). Levels of KDM5, HA and control histone H3 are shown. Eight imaginal discs were loaded per lane. * indicates non-specific band. (B) Comparison of the ability of untagged gKDM5 and gKDM5:HA to rescue kdm5K6801 mutants to viability. gKDM5 and gKDM5:HA rescue equally well. Results expressed as percent of expected progeny from the intercross of kdm5K6801/CyO; gKDM5 (or gKDM5:HA). At least 100 progeny were scored. ns = not significant. (C–G) ChIP analyses using anti-HA compared to IgG control in 3rd instar larvae. Promoters examined were 4E-BP, l(2)efl, CG5316, and Prx2540-2. InR is a Foxo-regulated gene that is not a KDM5 target so serves as a negative control. * indicates p<0.05. (TIF) [file pgen.1004676.s008.tif]

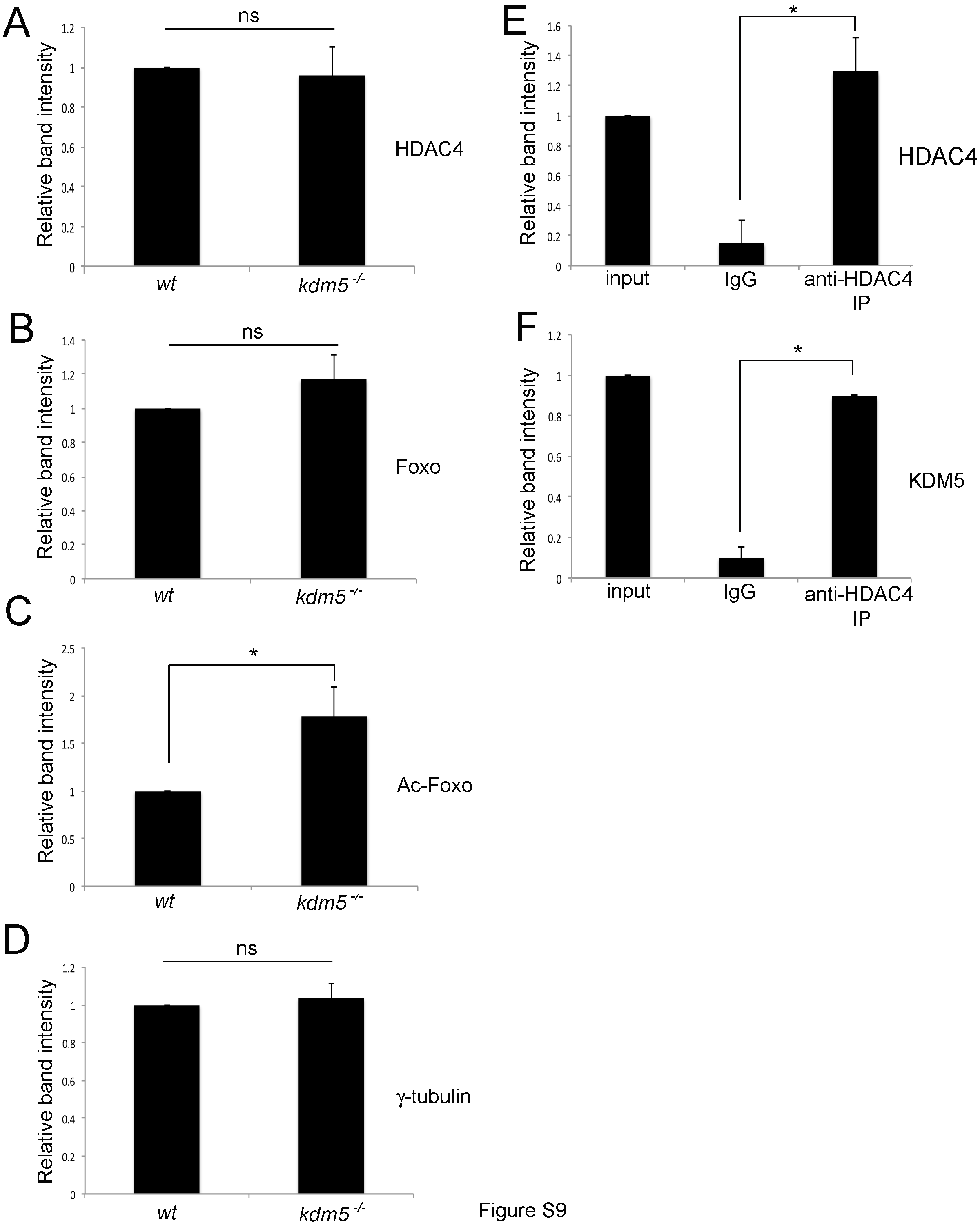

Supplement: Figure S9 — Quantitation of protein levels. (A–D) Quantitation of levels of HDAC4, Foxo, Acetylated Foxo and γ-tubulin (control) in wildtype (w1118) and kdm5K6801 homozygous larvae from three independent experiments as determined using LiCOR software. Protein levels are expressed relative to levels observed in wildtype. *p<0.05. (E, F) Quantitation of levels of HDAC4 and KDM5 in samples used for co-immunoprecipitation analyses. Protein levels are shown relative to levels observed in 10% input lane. * p<0.01. (TIF) [file pgen.1004676.s009.tif]

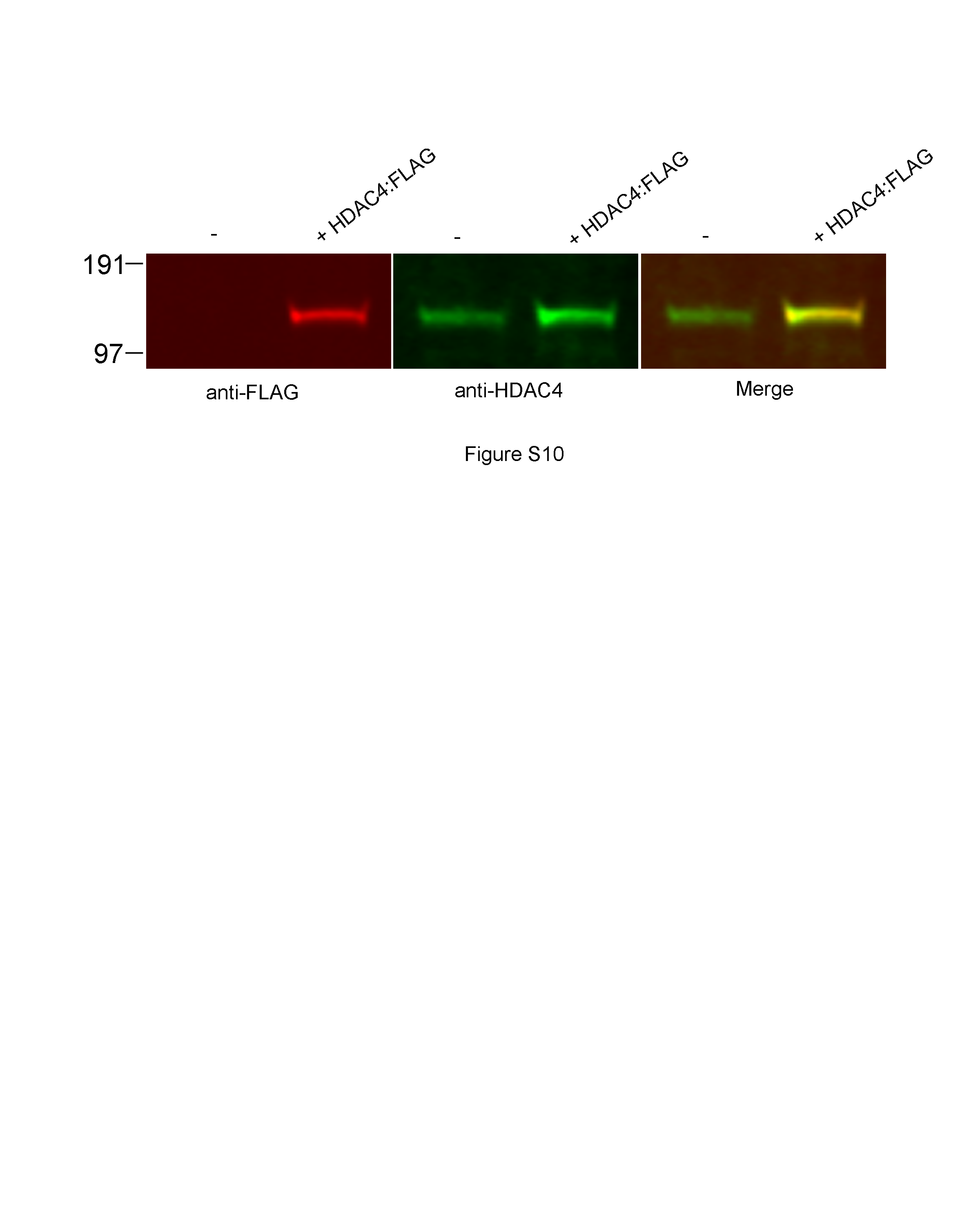

Supplement: Figure S10 — Specificity of HDAC4 antibody. Western blot analyses of S2 cell extract mock transfected (−) or transfected with a HDAC4:FLAG construct using anti-FLAG (left), an antibody that recognizes human HDAC4, 5, and 9 (middle) and a merge of the two channels (right). The co-migration of endogenous HDAC4 and FLAG-tagged HDAC4 shows that this antibody is specific to HDAC4 in Drosophila. (TIF) [file pgen.1004676.s010.tif]
