# Supplementary material for: KDM5 Interacts with Foxo to Modulate Cellular Levels of Oxidative Stress
Source: PLoS Genet. 2014 Oct 16;10(10):e1004676. doi: 10.1371/journal.pgen.1004676 (PMC4199495; doi:10.1371/journal.pgen.1004676)
Supplement: Table S1 — Adult survival of kdm5 alleles. (PDF) [file pgen.1004676.s011.pdf]

Table S1: Adult survival of *kdm5* alleles.

***kdm5* allele combination**	**number of *kdm5* mutants**	**total adult flies**	**Expected number**	**% expected**	***p* value (Chi squared)**
kdm5 [10424/10424]; +/+	3	650	216	1.40%	< 0.001
kdm5 [10424/10424]; gKDM5/+	40	110	37	108%	ns
kdm5 [K06801/K06801]; +/+	0	300	100	0%	< 0.001
kdm5 [K06801/K06801]; gKDM5/+	39	125	42	93%	ns
kdm5 [10424/K06801]; +/+	87	440	147	59%	< 0.001
kdm5 [10424/K06801]; gKDM5/+	70	200	67	104%	ns

Crosses were carried out to generate the genotypes indicated in the left-most column.
