# Supplementary material for: KDM5 Interacts with Foxo to Modulate Cellular Levels of Oxidative Stress
Source: PLoS Genet. 2014 Oct 16;10(10):e1004676. doi: 10.1371/journal.pgen.1004676 (PMC4199495; doi:10.1371/journal.pgen.1004676)
Supplement: Table S2 — Primers used for real-time PCR and ChIP. (PDF) [file pgen.1004676.s012.pdf]

Table S2: Primers used in this study.

RT-PCR primers	
Rp49 fw	CAGGCCCAAGATCGTGAAGAA
Rp49 RV	TGCGCTTGTTCGATCCGTAA
cyp4e2 FW	CTGCTGCTGGTTGCATATCT
cyp4e2 RV	ATTTCTGACGGATTCTTGCC
CG10211 FW	AACTCATTGAGGCTGCTGTG
CG10211 RV	GCCACACTTCGTACTCGAAA
GSTE1 FW	CAAGTATGCCAAGTCGGATG
GSTE1 RV	CACTGGCATCGAAGAAGAGA
4E-BP FW	CTTATCTACGAGCGGGCTTT
4E-BP RV	GAAGGGAGTACGCGGAGTT
l(2)efl FW	CTTCTCCTCATGCTTTCCCT
l(2)efl RV	CAACATCGACAGCGAGAAGT
Rala FW	GTTTCCTTTGCGTCTTCTCC
Rala RV	CCGCAAGATTTGTTCTCTGA
Gadd45 FW	GAACTGGACCTGGAGCTAGAGA
Gadd45 RV	AAAAGGCCTCCAGCAGTACCT
pr-set7 FW	CAACAAAGCAGGCATGAAAA
pr-set7 RV	GTGGCGTACTTGGAGACCAT
mthl1 FW	TCTCTCGATCTTCGCCTACTTC
mthl1 RV	GTGACACCCATAACGACGACTA
Glaz FW	GAACTTCGCCATCTTGTGGT
Glaz RV	GGGCATTGTTTGTTTTTGCT
CG3714FW	ACTGCAACAGCAACAGGAAC
CG3714RV	TGAAATTCAGCAAGCCACTC
spirit FW	CCCTTATCGCCAACAACTTT
spirit RV	GCAGCGCAATATCGTTGTAT
Nox FW	GCGGGAGTCATGGAGATATT
Nox RV	ATGATGGACTGCAGAATGGA
plod FW	ACCGATACGAAGAAGGGATG
plod RV	ACGGATCGATGAAGGAAATC
CG33099FW	ACCATGGGATAGCAGAGGAC
CG33099RV	GCAAACATTGTAGGCGTGAC
Prx2540-1 FW	GACGAGGAGCAGAAGAAGGA
Prx2540-1 RV	AGGGAGTCAATGGTCCTCAG
Prx2540-2 FW	AAGAGCTATTGCCTGGACATTC
Prx2540-2 RV	GGAGTCAATGGTCCTCAGAATC
Xbp1 FW	GACCATCTAACCTGGGAGGA
Xbp1 RV	TTTCCGTTCTGTCTGTCAGC
Gyc-89daFW	TTGAGTTCGAGTTGATTCGC

Gyc-89da RV	CCATAGTTTGGGCTTCGTTT
Gkt FW	GGGATACTGGACAAGCCACT
Gkt RV	CGAGGAACATCATCTTGGTG
CG5316 FW	AATCGCAGTGGTCATAGCAG
CG5316 RV	GGAGACGGAAGCACAATCTT
CG12896 FW	CTCTCCATGTTTTACCCCATGT
CG12896RV	AAATCCCTTGGGGAAGAGTTTA
InR FW	GCGATGATCGCTGGAAGGTT
InR RV	GCGATGATCGCTGGAAGG TT
HDAC4_FW	TCCCGAGGTCAAACAGATTC
HDAC4_RV	GCGGCTGAGGTATTTTGTA

ChIP primers

4E-BP promoter FW	GAATGCGATTGGCGTTTAGT
4E-BP promoter RV	CTCGCCTTGAGCTCTTGTTT
CG5316 promoter FW	CATGCACAGAGGTAAACAACAGT
CG5316 promoter RV	GGATGCCGATATGAGGGTTA
sprit promoter fw	TTCAATGAGCCAAATCCAAG
sprit promoter rv	TTTGATAGCAGCCAGAATCG
Prx2540-2 promoter FW	TCACACACATGACACCATGC
Prx2540-2 promoter RV	AATAGCGGGTGCGATAGAGA
InR-RA promoter FW	TGGTTTAAATTGGTTTTACAGCAG
InR-RA promoter RV	CATGCAATCAAGCATAATCATTT
puc promoter FW	TCC CTC TCT CTT GCT CTC TTG
puc promoter RV	AAT GAA ATG GAG ACG CTT ACG
CG10211 promoter FW	CCTGTCCCATGCTGTGTAGTT
CG10211 promoter RV	GACTCTCTTTTGGCCCACAC
l(2)efl promoter FW	CGTGGGGGAAGATGTAAACAA
l(2)efl promoter FW	CTCGAGCACTCACACGATTTTA
4E-BP FW -1322	CTG TCC CAC ATC GAG ATC AT
4E-BP RV -1129	ACG ATT CGA CGT AAT TCG G
l(2)efl Fw -981	TTT AGG GAC TGG CCC TGT AG
l(2)efl RV -915	GGT GAT TGC CTC ACT GTT TG
spirit FW +3594	CAG CAT CTG GAT CTT GCA GT
spirit RV+3694	CAA GCA AAT GCC GGA TAA C
CG10211 FW +14704	TCT GCA ACT GTG TAC GAG CA
CG10211 RV+14798	ACC CAA ACC ATC TTG GTA GC
CG5316 FW+2423	CAA CAT TCA AGG CAA CAT CC
CG5316 RV+2570	CAT ATC GAT GGA GCT GTC GT
prx2540 FW +1475	ATA TCC GAG TCG AGG CTG AG
prx2540-2 RV +1567	GTG CGA ACG AGA AAG TTC AA
